# TF-MAPS: fast high-resolution functional and allosteric mapping of DNA-binding proteins

**DOI:** 10.1038/s41594-026-01908-2

**Published:** 2026-11-11

**Authors:** Xianghua Li, Ben Lehner

**Affiliations:** 1 Wellcome Sanger Institute, Cambridge, UK; 2 King’s College London, London, UK; 3 Centre for Genomic Regulation (CRG), Barcelona Institute for Science and Technology (BIST), Barcelona, Spain; 4 Universitat Pompeu Fabra (UPF), Barcelona, Spain; 5 Institució Catalana de Recerca i Estudis Avançats (ICREA), Barcelona, Spain

**Keywords:** Biological techniques, Biophysics, Genomics, Drug discovery

## Abstract

Transcription factors (TFs) bind specific DNA sequences to control gene expression. Modulating TF activity is of considerable therapeutic interest but very few TFs have been drugged successfully. TF DNA-binding interfaces are large and most TFs have no known allosteric ligands or allosteric sites. Here we introduce TF-MAPs, a scalable and general platform to chart complete functional maps of sequence-specific DNA-binding proteins. We apply TF-MAPS to three human disease TFs: HNF1A, FOXG1 and FOXP1. Allostery in all three proteins is distance-dependent, anisotropic and couples the DNA-binding interface to the protein surface. The allosteric surfaces of FOXG1 and FOXP1 are quite diverged, revealing a potential for protein-specific regulation in protein families. All three TFs have potentially druggable small pockets where mutations affect DNA binding, either within the binding interface or distant from it. Application of TF-MAPS will potentially lead to charting functional and allosteric maps for hundreds of human TFs and other DNA-binding proteins to understand DNA recognition and TF regulation, to interpret clinical variants mechanistically and to guide the development of TF-modulating therapeutics.

## Abstract (web summary)

Li and Lehner present TF-MAPS, a scalable platform to chart functional maps of sequence-specific DNA-binding proteins. The authors map allostery in three transcription factors implicated in human disease and reveal potentially druggable small pockets.

## Main

The human genome encodes >1,500 TFs—sequence-specific DNA-binding proteins that regulate gene expression. The DNA-binding specificities of hundreds of TFs have been quantified alone^1^ and in combination^2^, as has their binding across the genome in several cell types^3^. Many TFs are high-value therapeutic targets. Rare variants in 164 TFs cause 277 human genetic diseases (ClinVar 11 November 2023, https://www.clinicalgenome.org/data-sharing/clinvar/) and many TFs are implicated in common diseases^4–6^. In addition, at least 190 TFs are encoded by cancer driver genes^4,7^, including 87 oncogenes and 60 tumor suppressors^8–10^.

Despite their causal role in hundreds of human diseases, TFs are considered very challenging therapeutic targets. TFs have no catalytic sites to inhibit using small molecules. Moreover, they bind DNA through charged interfaces larger than drug-sized small molecules^11,12^. For many TFs, such as tumor suppressor genes, the desired therapeutic outcome is activation, not inhibition^13^. For destabilizing missense variants, small-molecule stabilizers (also known as correctors or pharmacochaperones) may present a viable therapeutic strategy, and correctors have been developed to bind and stabilize p53^14^. In addition, the serendipitous discovery of molecular glues that recruit ubiquitin ligases to degrade oncogenic TFs has led to extensive efforts to develop new TF degrader therapeutics^15^. However, the vast majority of TFs mutated or implicated in human diseases have not been targeted therapeutically^11^.

One class of human TFs that has been drugged very successfully is nuclear receptors (NRs). NRs are a family of 48 human TFs activated or inhibited by binding of small molecules such as hormones. Examples include the estrogen, progesterone, androgen and steroid hormone receptors. Ligands bind these TFs at a site distant to the DNA-binding interface and indirectly alter the binding of the receptors to other proteins, DNA and nuclear localization by causing changes in conformation and dynamics^16^. This mechanism of regulation is an example of allostery—the long-range transfer of information between distant sites in proteins^17^. Over one-third of NRs have been drugged successfully, with nearly 200 licensed inhibitors and activators approved by regulatory agencies^18^. Indeed, NRs are the third most successfully drugged protein family after G protein-coupled receptors and ion channels, demonstrating the huge potential for TF-targeting therapeutics^19^. Another example is the bHLH–PAS (basic helix–loop–helix–PER–ARNT–SIM) family and small-molecule inhibitors have been developed successfully against hypoxia-inducible factor 2α^20^, the aryl hydrocarbon receptor^21^ and the master circadian regulator brain and muscle ARNT-like protein 1^22^.

Allostery is central to biological regulation and the efficacy of many licensed drugs^23^. However, most proteins, including most TFs, have no known allosteric sites^24^. Recently, powerful approaches have been developed to quantify allosteric communication systematically and comprehensively in proteins using mutations^25–28^. The general approach involves quantifying the effects of mutations in all sites in a protein on a selectable activity and also quantifying the effects of the same mutations on the concentration of the folded protein. Combining the two selections^25,29,30^ allows the effects of mutations on activity not caused by changes in the concentration of the folded protein to be quantified. By definition, if a mutation outside of an active site alters activity or binding, it must be through an indirect or allosteric mechanism^25–27^.

These complete experimental allosteric maps have revealed that allostery is surprisingly pervasive. These allosteric maps share a characteristic, approximately exponential, distance-dependent decay of allosteric mutational effects away from the active site of the protein^25–28,31,32^. Allostery is, however, anisotropic, with mutations in particular sites having stronger allosteric effects than other mutations at the same distance from the active site. We refer to these sites as ‘allosteric hotspots’^28^. This spatial and functional mapping of allosteric effects provides a powerful framework for identifying therapeutically actionable sites^26,27^.

Here we present a general and fast experimental platform that extends allosteric mapping to DNA-binding proteins, including TFs. We first use the platform to comprehensively map single amino acid substitutions in the POU-homeodomain (POU_H_) DNA-binding domain (DBD) of hepatocyte nuclear factor 1 homeobox alpha (HNF1A)—a master regulator of gene expression in the liver, pancreas, kidneys and intestines^33^. Autosomal dominant variants in HNF1A cause maturity-onset diabetes of the young type 3 (MODY3)—a condition characterized by reduced insulin secretion due to pancreatic β-cell dysfunction^33^; common single-nucleotide polymorphisms in HNF1A are associated with increased risk of type 2 diabetes mellitus^34–39^. Somatic and germline variants in HNF1A also cause hepatic adenoma and carcinoma^40,41^, with ~35% of hepatocellular adenoma cases attributed to inactivating HNF1A mutations^40,41^. Gain-of-function mutations in HNF1A protect against diabetes but increase hepatically derived lipoprotein risk factors for atherosclerosis and coronary artery disease^42^. No licensed drugs target HNF1A. HNF1A binds DNA through two domains: POU_H_ and a POU-specific domain (POU_S_); both harbor pathogenic variants in MODY3, but only the POU_H_ is mutated frequently in hepatocellular adenoma^43^.

We next apply TF-MAPS to a distinct TF family and generate comparative functional maps for two forkhead box (FOX) TFs, FOXG1 and FOXP1 DBDs. FOXG1 and FOXP1 have the largest number of known pathogenic variants among the 43 human forkhead family TFs^44,45^. Both TFs function during development, and loss-of-function mutations cause rare severe neurodevelopmental disorders called FOXG1 syndrome and FOXP1 syndrome, respectively^46,47^. FOXP1 is important for immune regulation and has been characterized as a potential tumor suppressor in various malignancies^48,49^. Duplications of FOXG1 and FOXP1 are also pathogenic: FOXG1 duplications cause a distinct neural pathology from loss‑of‑function mutations^46,50^, whereas FOXP1 duplications promote oncogenesis in lymphomas and other carcinomas^51^.

The functional and allosteric maps of these three DBDs provide insights into DNA recognition, fold stability, long-range energetic coupling and functional pockets. Using the TF-MAPS approach, it should be possible to produce functional and allosteric maps for hundreds of human TFs to understand DNA recognition, interpret clinical variants mechanistically and guide the development of allosteric therapeutics to modulate TF DNA-binding.

## Results

### TF-MAPS: rapid functional and allosteric mapping for DNA-binding proteins

We conceived a general approach called TF-mutational allosteric propagation scan (TF-MAPS) to comprehensively quantify residue function and allostery in DNA-binding proteins. TF-MAPS tests whether perturbations at every site in a protein alter its binding to DNA directly or indirectly.

**Fig. 1:**
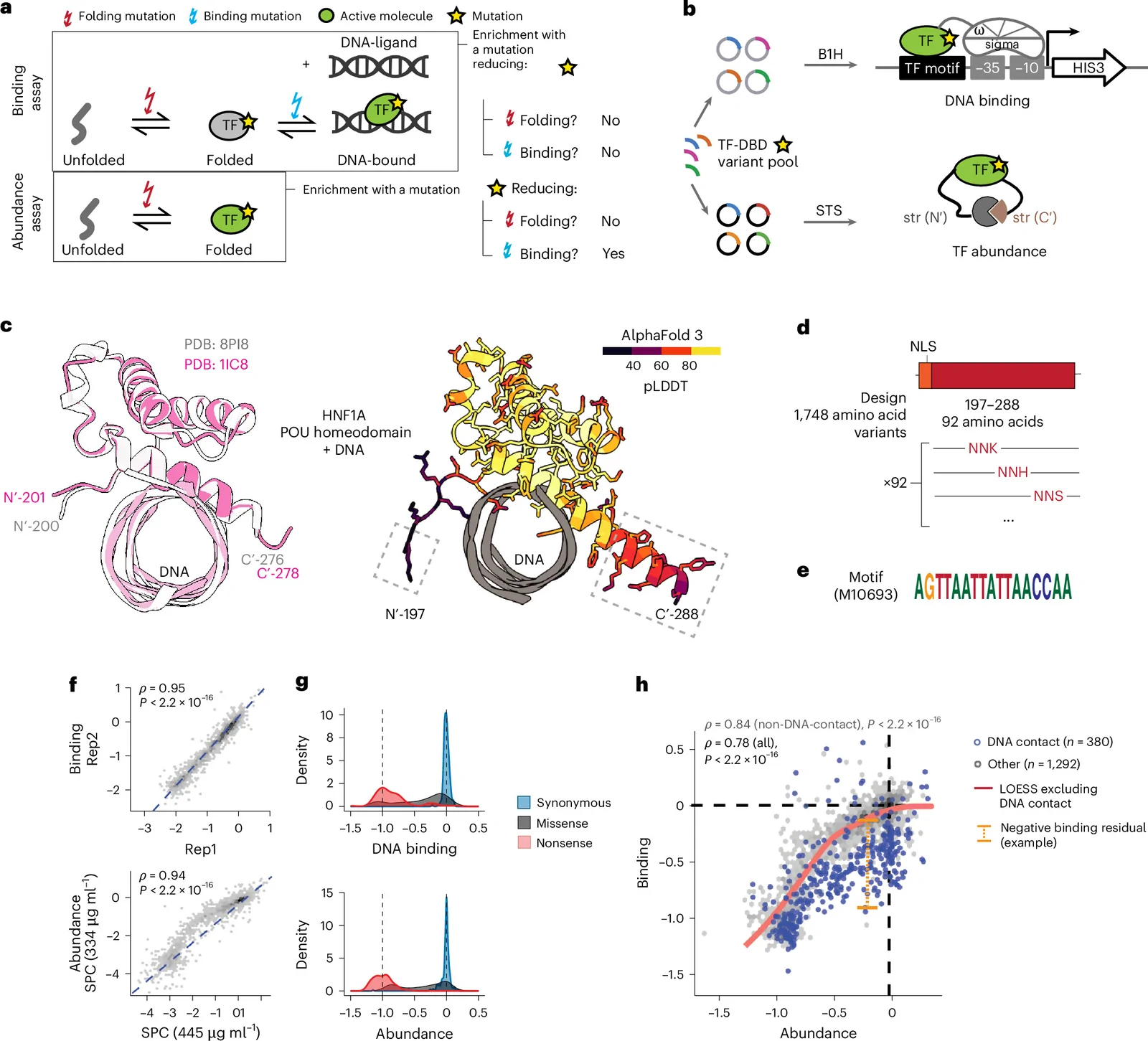
TF-mutational allosteric propagation scan. **a**, Schematic overview of mutational effects assessed by DNA-binding and protein abundance assays, illustrating their impact on TF states. **b**, Reporter systems used in the experiments: B1H assay for DNA-binding activity, and STS assay for protein abundance. **c**, Structures of HNF1A POU_H_ bound to DNA. Left: experimentally solved; right: AlphaFold 3-predicted. Dashed boxes highlight regions absent in the solved structure. **d**, Composition of the mutant library targeting HNF1A POU_H_ and flanking regions. Degenerate codons: N = A/T/G/C; K = G/T when WT is C or A; S = C/G when WT is A; H = A/C/T when WT is G. **e**, HNF1A binding motif used in the DNA-binding assay. **f**, Assay reproducibility and mutation distributions before scaling mutational effects between 0 and −1. DNA-binding assay shows correlation between biological replicates; abundance assay shows correlation across two conditions. SPC, spectinomycin. **g**, Distribution of mutations across functional classes after normalization (0 = WT; −1 = mode of nonsense mutations). **h**, Relationship between abundance and binding effects, with residuals computed from a LOESS fit on non-DNA-contacting residues. Statistical significance in **f** and **h** was determined using a two-sided Spearman’s rank correlation coefficient test with a large-sample normal approximation. No adjustments were made for multiple comparisons. Source data

TF-MAPS has two experimental steps. First, the effects of all mutations in all sites on the protein’s ability to bind to a specific DNA sequence are quantified. Second, the effects of the same mutations on the folded protein abundance are quantified. Integrating these two measurements then allows quantifying changes in binding beyond those caused by changes in concentration of the folded protein to be determined (Fig. 1a).

To quantify DNA binding, TF-MAPS uses a modified bacterial one-hybrid (B1H) assay, which uses a fusion between the TF of interest and a subunit of RNA polymerase^52–54^, enabling the protein interaction with a target DNA sequence to regulate expression of a reporter gene (HIS3). The level of reporter gene expression directly influences the cellular growth rate, thereby providing a quantitative measure of DNA-binding activity (Fig. 1b).

To quantify folded protein abundance in the same cellular context of *Escherichia* *coli*, TF-MAPS uses a spectinomycin tripartite sandwich (STS) assay^55^. Insertion of the DBD into a split streptomycin-resistance protein links bacterial growth to TF folding, as only the folded protein confers antibiotic resistance^55,56^.

The TF-MAPS approach is inspired by methods developed for producing allosteric maps of protein–protein interaction domains^25,26,28^, kinases^27^ and receptors^32,57,58^. However, because TF-MAPS is conducted entirely in bacteria, it offers the advantages of speed, simplicity and small volumes.

### Functional map of HNF1A POU_H_

**Extended Data Fig. 1:**
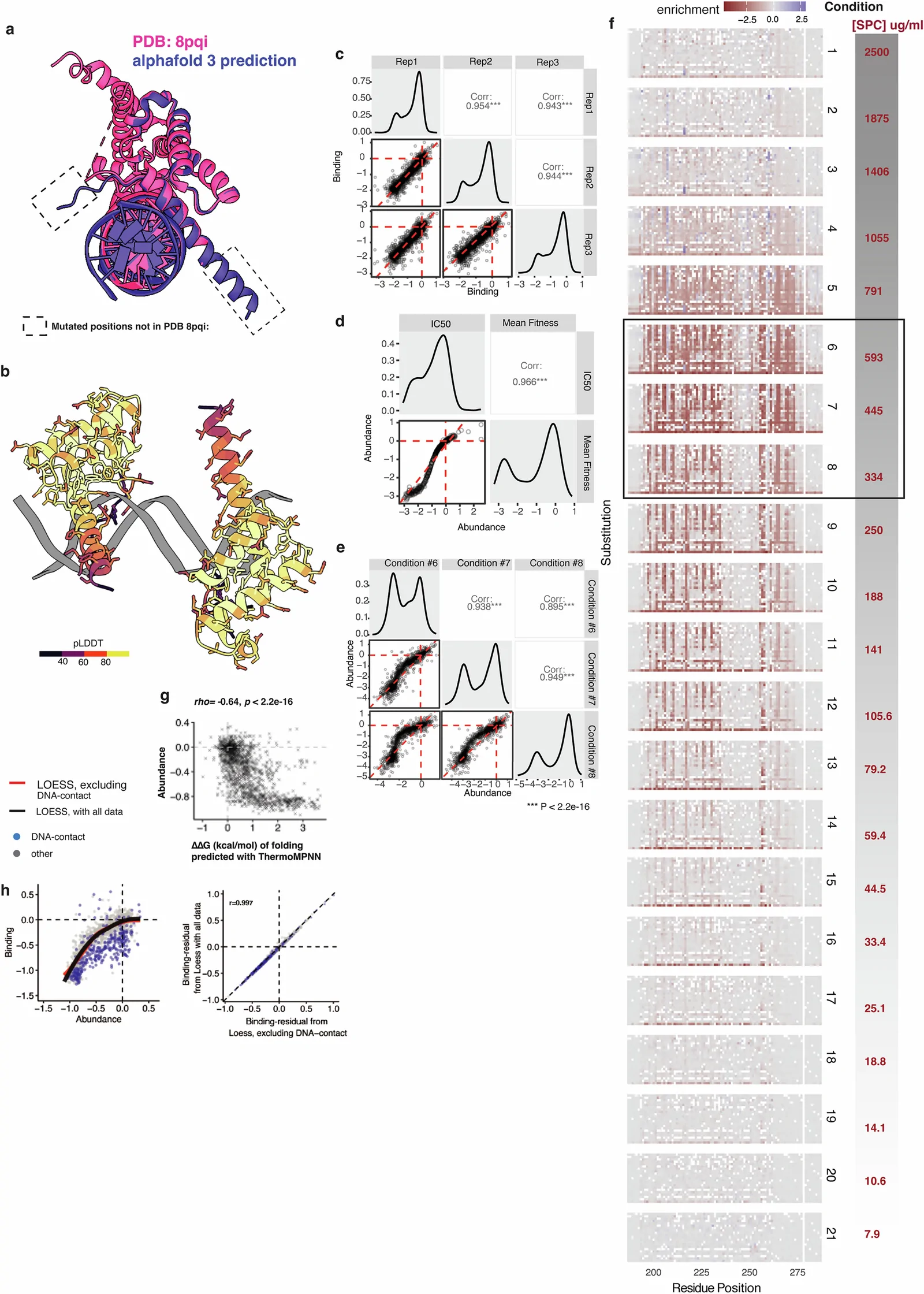
Reproducibility of the experiments with HNF1A POU_H_. (**a**) Overlay of the AlphaFold3-predicted DNA-bound HNF1A POU_H_ region and the experimentally solved DNA-bound structure of the HNF1A DNA-binding domain. (**b**) AlphaFold3-predicted HNF1A POU_H_ two monomers bound to the palindromic sequence of DNA. (**c–e**) Correlation matrices showing assay reproducibility for the binding assay (**c**) and abundance assay (**d, e**). Weighted means of abundance scores in (**d**) were taken from conditions #6, 7, and 8, which are shown individually in (**e**). (**f**) Heatmaps of the abundance assay across all 21 tested conditions with different concentrations of spectinomycin. Conditions #6,7,8 are highlighted. (**g**) Comparison of STS assay results with ThermoMPNN-predicted protein stability changes. (**h**) Comparisons of the LOESS fit (left) and binding residuals (right) between conditions with and without DNA-contacting residues. Statistical significance was determined using a two-sided Spearman’s rank correlation coefficient test with a large-sample normal approximation. No adjustments were made for multiple comparisons. Source data

We first applied TF-MAPS to an important human disease TF DBD—the POU_H_ from HNF1A (Fig. 1c and Extended Data Fig. 1a,b), mutations in which cause MODY3^33^ and hepatocellular adenoma^43^.

We constructed a library containing all 19 amino acid substitutions, stop and synonymous variants in all sites in the DBD (residues 197 to 288) (Fig. 1d). We then quantified binding to a consensus DNA motif using the B1H system, and the folded protein abundance using STS (Fig. 1b,e,f and Extended Data Fig. 1c–g; median Spearman’s *ρ* = 0.944 for B1H replicates, *ρ* = 0.938 for STS at three concentrations).

**Fig. 2:**
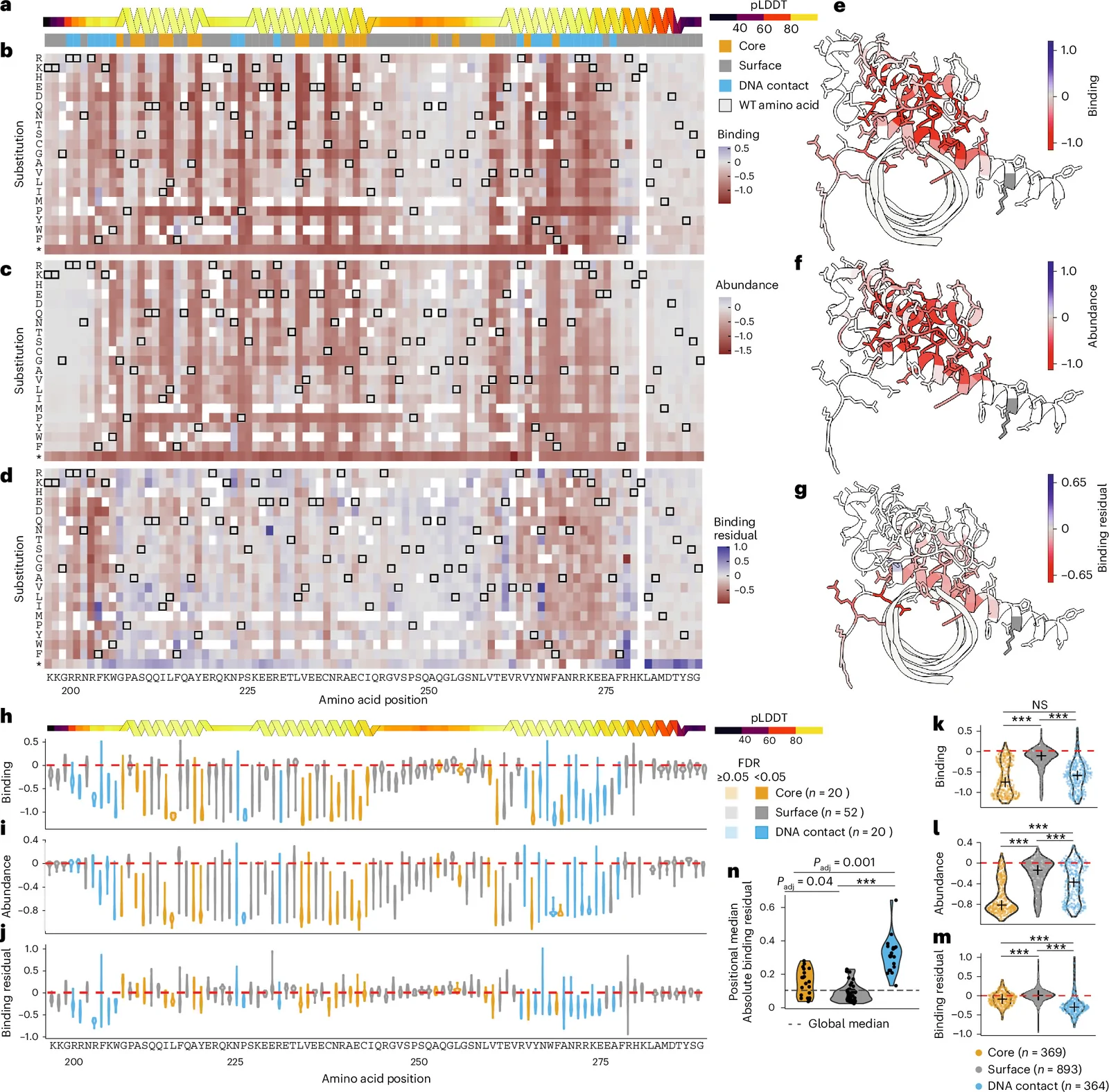
Functional map of HNF1A POU_H_ DBD. **a**, Structural annotation of residues based on the AlphaFold 3-predicted DNA-bound structure. **b–d**, Heatmaps showing mutational effects from the DNA-binding assay (**b**), protein abundance assay (**c**) and binding residuals (**d**), with missing values left blank. **e–g**, Median mutational effects per amino acid residue for DNA binding (**e**), abundance (**f**) and binding residuals (**g**). The position without data is shown in dark gray. **h–j**, Violin plots of positional median effects for DNA binding (**h**), abundance (**i**) and binding residuals (**j**). Statistical significance was assessed using one-way Wilcoxon tests followed by BH FDR correction. **k–m**, Comparison of mutational effects across three structural classes (core, surface and DNA-contacting residues) for DNA binding (**k**), abundance (**l**) and binding residuals (**m**). NS, non-significant. Statistical significance was assessed using Kruskal–Wallis tests followed by post hoc Dunn’s tests with Bonferroni correction. Adjusted *P* values (*P*_adj_) for comparisons between core and DNA-binding residues, core and surface residues, and surface and DNA-binding residues are as follows: 1.0, 4.28 × 10^−75^ and 3.1 × 10^−71^ (**k**); 2.38 × 10^−20^, 1.49 × 10^−72^;and 8.6 × 10^−12^ (**n**) and 5.95 × 10^−27^, 7.73 × 10^−16^ and 5.63 × 10^−19^ (**m**). **n**, Comparison of positional median absolute binding residuals across the three structural classes. Kruskal–Wallis test followed by post hoc Dunn’s test with Bonferroni correction were used to calculate *P* values. ***P_adj_ = 6.09 × 10^−11^. Source data

**Extended Data Fig. 2:**
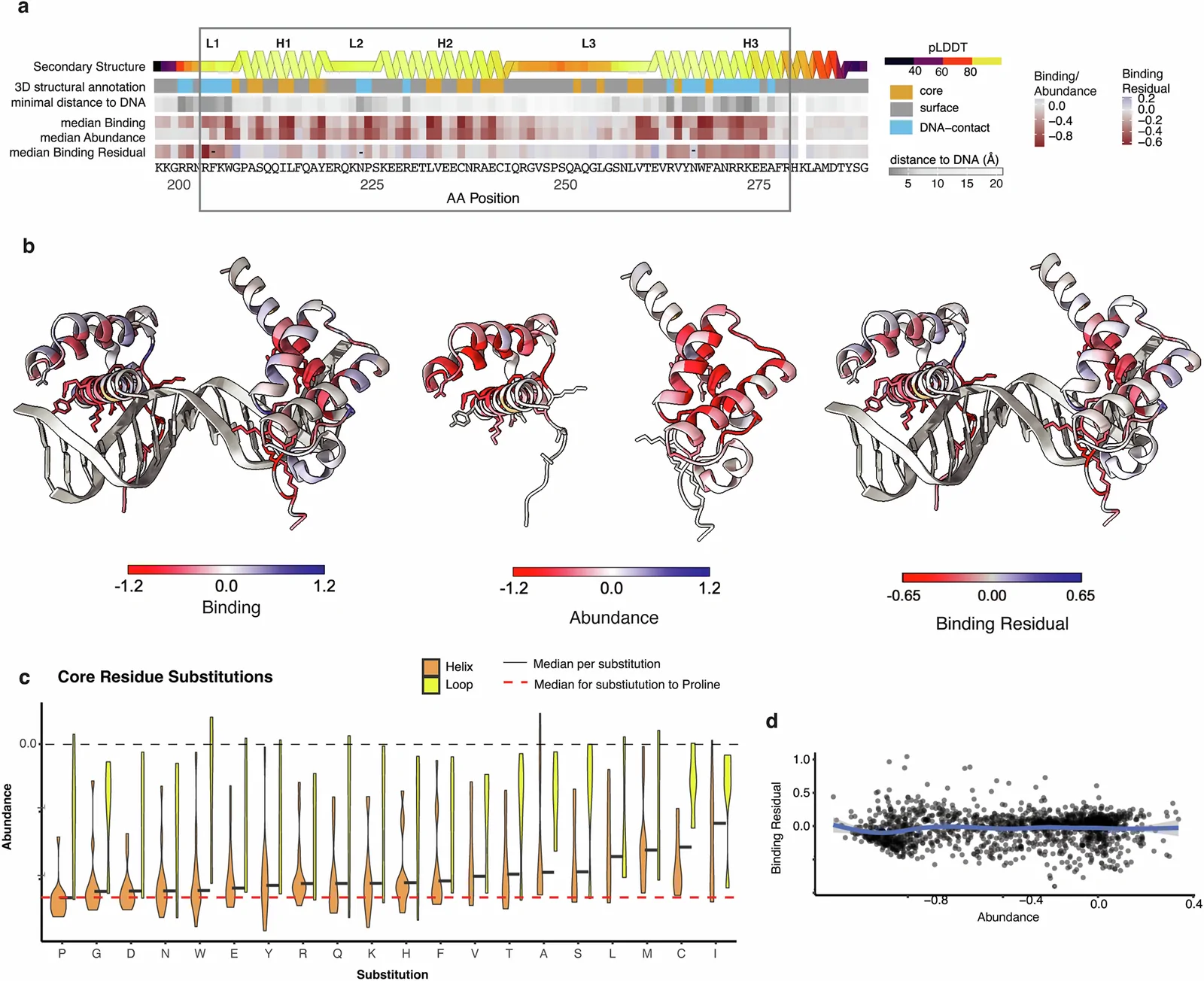
Distribution of HNF1A POU_H_ mutational effects on DNA binding and protein abundance. (**a**) Structural annotations and their positional median mutational effects, with the boxed region representing the conserved POU_H_ annotation. (**b**) Structural view of the median mutational effects, with the side-chain atoms shown only for the DNA-contacting residues (**b**). (**c**) Comparison of abundance effects by substitutions to each amino acid. (**d**) Scatter plot showing the relationship between binding residuals and abundance for all missense mutations. The blue line represents the LOESS fit, and the grey shading indicates the corresponding 95% confidence interval.

Plotting the binding of each HNF1A variant against its abundance reveals a strong correlation (Spearman’s *ρ* = 0.78; Fig.1h), consistent with many changes in binding being caused by changes in abundance^25,26,28^. As expected, mutations in the hydrophobic core are very detrimental for abundance (median abundance = −0.82) (Fig.2a,c,f,i,l and Extended Data Fig. 2a,b), with substitutions to proline most detrimental (median = −0.91), particularly in secondary structure elements (median = −0.95) (Fig. 2a,c,f,i,l and Extended Data Fig. 2c). Also as expected, mutations in the 20 residues that contact DNA (Methods) are highly detrimental for binding (median binding = −0.37 for all 20 residues and −0.45 for 12 residues in the structured helices), often without causing large changes in abundance (Fig. 2h–m).

To quantify the impact of each mutation on binding beyond the abundance change, we used locally estimated scatterplot smoothing (LOESS) regression to infer the expected change in binding for each variant, given its folded abundance^31,57^ (Methods). For each mutation, the binding residual between the measured and expected binding change quantifies its effect on binding beyond the change caused by folded abundance (Fig. 1h). These binding residuals are well-distributed across the dynamic range of abundance measurements (Extended Data Fig. 2d).

### The HNF1A–POU_H_ DNA interface

**Fig. 3:**
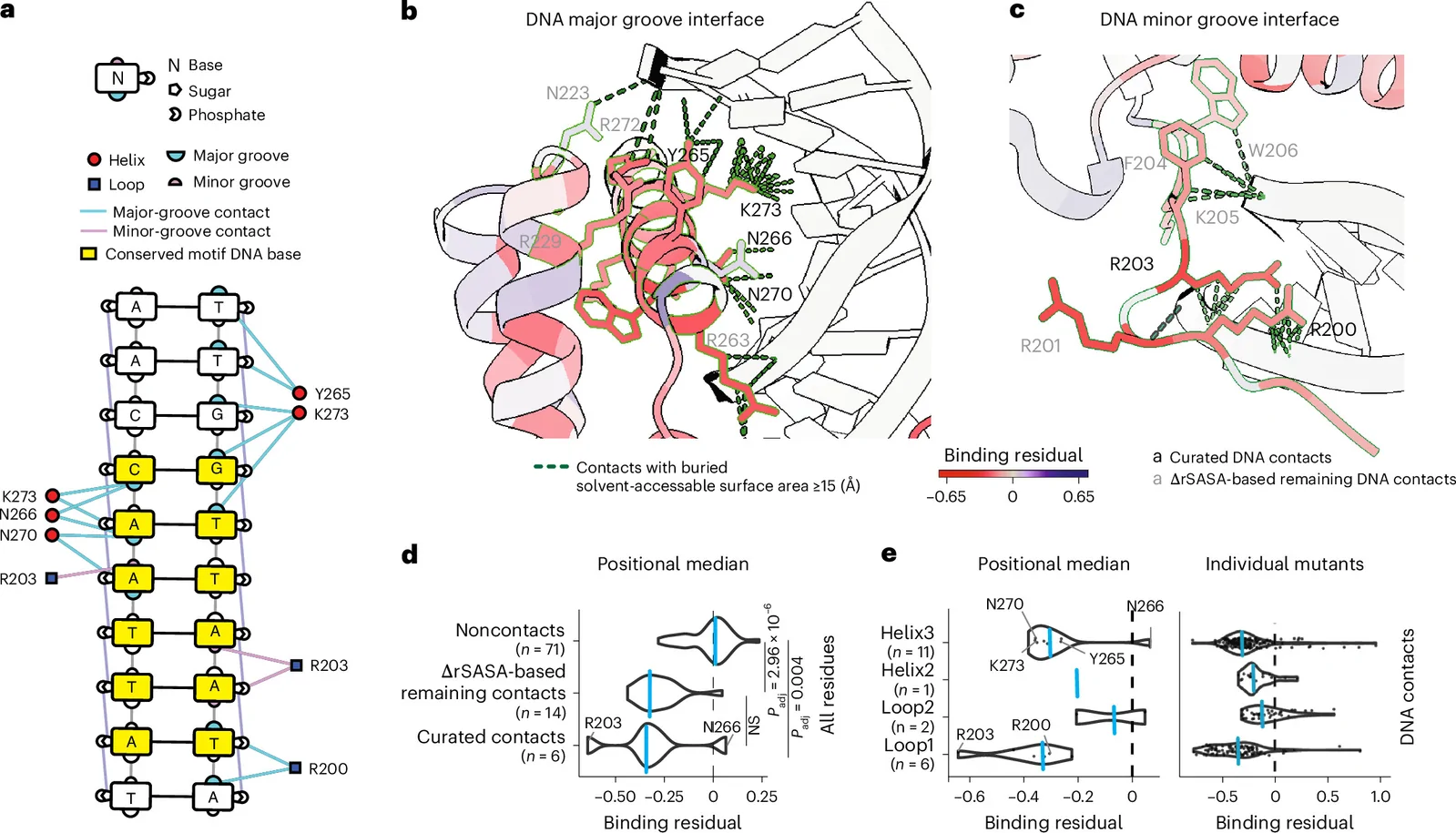
HNF1A–POU_H_ DNA-binding interface. **a**, DNA-contact map generated with DNAproDB based on the AlphaFold 3-predicted structure of the DNA-bound monomer. **b**,**c**, DNA-bound structure of HNF1A POU_H_ highlighting broadly defined DNA-contacting residues based on the ChimeraX ‘contacts’ function at the major (**b**) and minor (**c**) grooves. Side chains of contacting residues are shown in atomic detail; the rest of the protein is depicted as a backbone cartoon. Dashed lines: protein–DNA contacts. **d**,**e**, Mutational effects at residues classified as ∆rSASA-based DNA contacts (**d**) and secondary structures (**e**). Kruskal–Wallis test followed by post hoc Dunn’s test with Bonferroni correction was used to calculate *P*_adj_ in **d**. *P*_adj_ = 0.004 and 2.96 × 10^−16^. Source data

**Extended Data Fig. 3:**
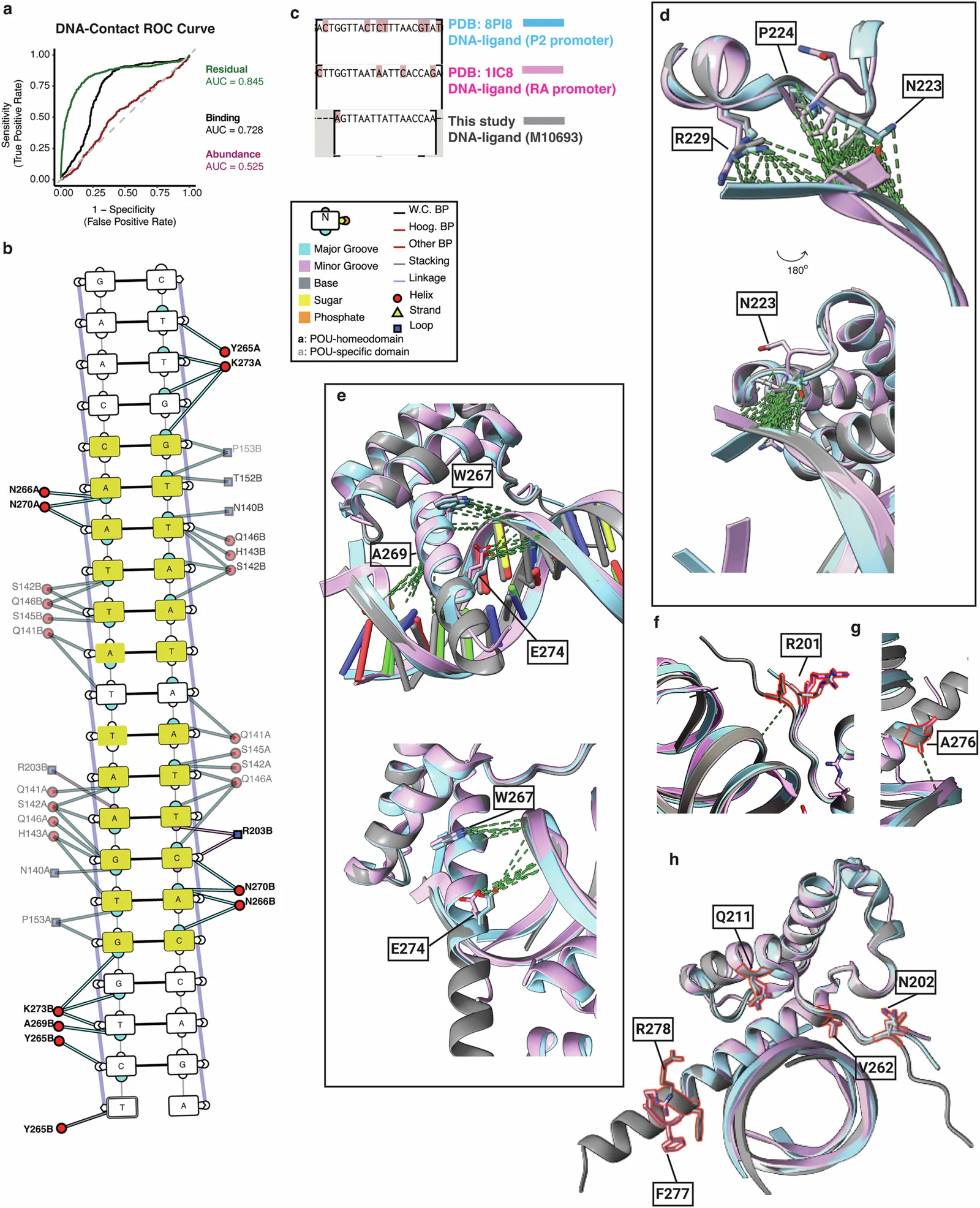
HNF1A POU_H_ DNA-binding interface. (**a**) ROC curves for classifying DNA-contacting residues using positional median binding-residuals, as well as binding and abundance scores. (**b**) Illustration of DNA-contacting residues based on PDB 1IC8. (**c**) Sequence alignment of DNA used in this study and in the two published structures. Non-identical bases are highlighted. (**d-h**) Three aligned structures of HNF1A POU_H_ bound to DNA, viewed from different angles highlighting different positions.

**Extended Data Fig. 4:**
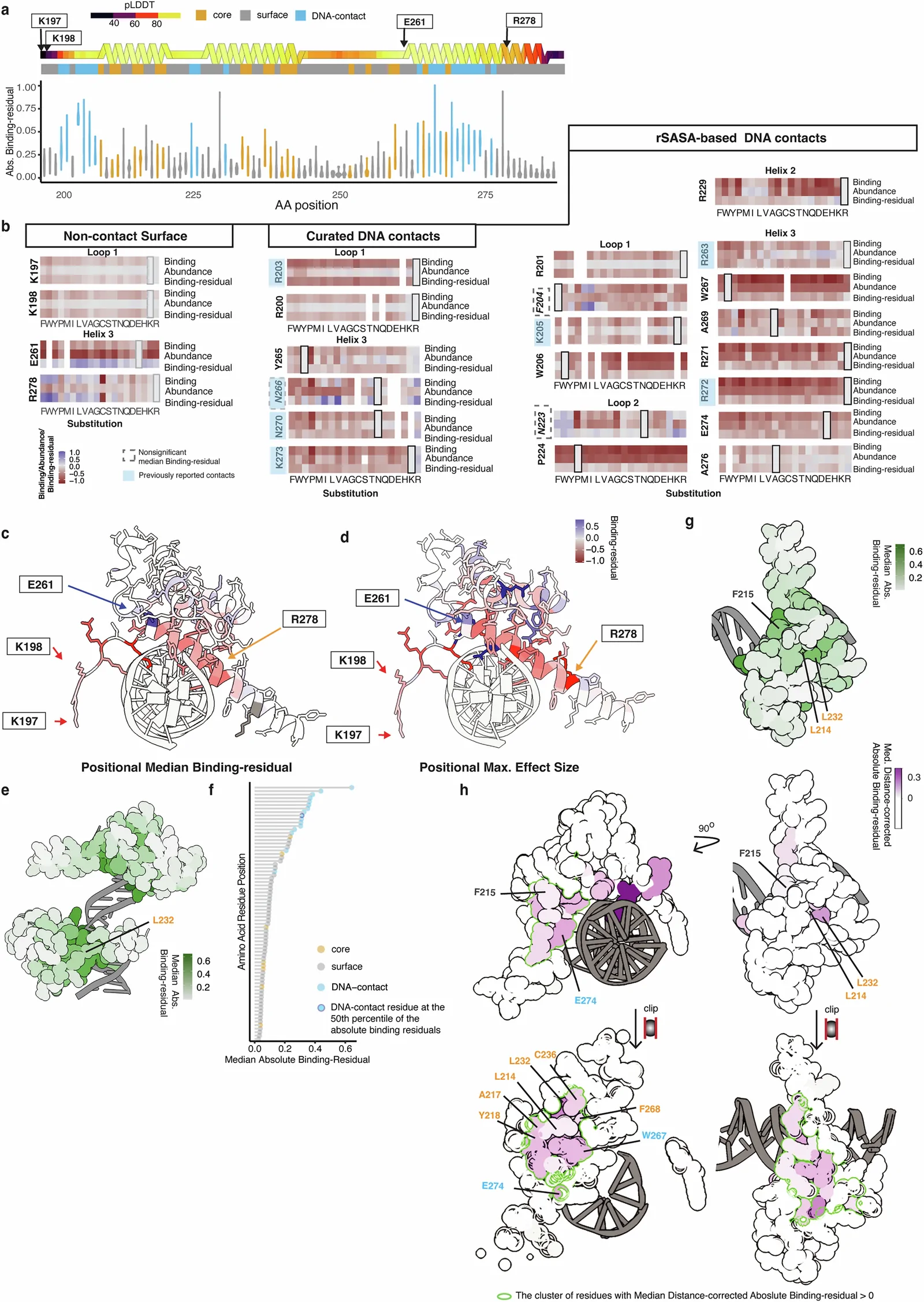
DNA-binding residuals of HNF1A POU_H_. (**a**) Absolute binding residuals plotted along the amino acid sequence of HNF1A POU_H_. (**b–d**) DNA-contacting positions and example surface-exposed positions showing large absolute residuals for all mutations (**b**), highlighted on positional medians (**c**) and maximum effect sizes (**d**), illustrated on 3D structural views. (**e**) Structural view of two HNF1A POU_H_ monomers bound to DNA, with absolute residuals colour-coded by magnitude. (**f**) Ordered positional median absolute binding residuals. (**g, h**) 3D view of the HNF1A POU_H_ bound to DNA, coloured by the positional median absolute binding residuals (**g**), and residuals after accounting for the distance-dependent decay (**h**).

As expected, many mutations in the DNA-contacting residues have large binding residuals (Fig. 2a,d,g,j,m and Extended Data Fig. 3a). However, not all residues that contact DNA are equally important for binding (Fig. 2j,m). Overall, mutations in Loop 1 of the POU_H_ domain produce the strongest negative effects on DNA binding, followed by those in Helix 3 (Fig. 3g). In contrast, mutations in Loop 2 and Helix 2 have smaller effects, suggesting a hierarchy of functional importance among DNA-contacting regions (Fig. 3). DNAproDB^59^ reports six residues (four in Helix 3: N266, N270, K273, Y264; and two in Loop 1: R200 and R201) with base-specific contacts with DNA in the Alphafold 3-predicted structure. We call these curated contacts to differentiate them from other contacts identified by changes in solvent accessibility (∆rSASA) (Fig. 3a and Extended Data Fig. 3). Several residues reported previously to contact DNA^60–62^ but not identified by DNAproDB, including R263 and R272 within Helix 3 and R203 and K205 within Loop 1, also contribute strongly to DNA binding. Mutations at residues in curated and ∆rSASA-based contacts similarly reduce binding residuals (Fig. 3b–d and Extended Data Fig. 4a). Overall, 11 residues within Helix 3 contact DNA, and mutations in 10 of these impair DNA binding, whereas mutations in the remaining residue, N266, both enhance and impair binding (Fig. 3b,e and Extended Data Fig. 4a,b).

### Mutational evidence for an extended binding interface

Interestingly, AlphaFold 3^63^ predicts three additional DNA-contacting residues, R200, R201 and A276, not in the experimental structures^60,64^. Residues 197–201 in the linker between the POU_S_ and POU_H_ domains (Loop 1) are unresolved in the experimental structures. They form part of the nuclear localization signal (NLS), and mutations in this region are proposed to be pathogenic because they disrupt nuclear localization^65^. AlphaFold 3^63^ predicts that two residues (R200 and R201) contact DNA directly and indeed mutations at these sites strongly impair DNA binding (Figs. 2a–j and 3a,c–e and Extended Data Figs.3f and 4b). Residue A276 is resolved as part of Helix 3 in one DNA-bound structure (PDB 8PI8)^64^ but in a loop in another DNA-bound structure (PDB 1IC8)^60^ (Extended Data Fig. 3g); it does not directly contact DNA in either structure. In the AlphaFold 3 model, however, A276 lies in an extended Helix 3 and makes a backbone hydrophobic contact with the nonconsensus region of the DNA ligand (Ala Cβ—thymine’s C7 methyl, 4.773 Å) (Extended Data Fig. 3g). Consistent with this, mutations at A276 strongly reduce DNA binding (Extended Data Fig.4a,b).

### Distance-dependent allosteric decay

**Fig. 4:**
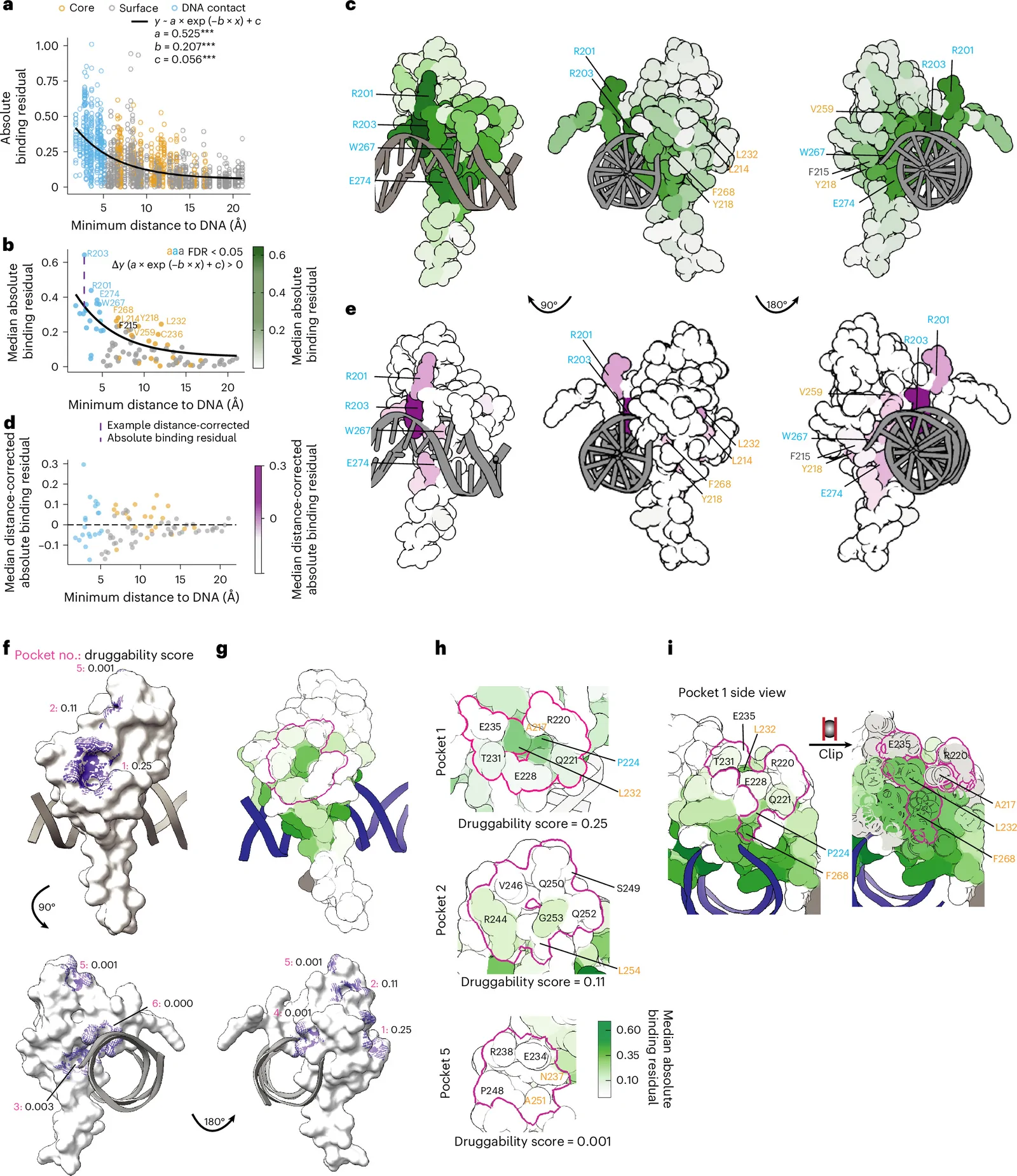
Allosteric regulation of DNA-binding in HNF1A POU_H_ domain and pocket prioritization. **a**,**b**, Distance-dependent decay plots of absolute binding-residuals for all mutations (**a**) and positional medians (**b**), colored by structural annotation. **c**, Structural views of absolute binding residuals from several perspectives, highlighting positions with significantly elevated residuals compared to decay-predicted values (FDR < 0.05, Wilcoxon test). **d**, Residuals of DNA binding after fitting a decay curve based on distance to DNA. **e**, Structural views of allosteric anisotropy from different angles. **f**, All pockets identified by the FPocket algorithm shown on the DNA-bound structure from several perspectives; pocket-forming residues are shaded. **g**, Pocket 1 residues viewed from the opposite side of DNA, encircled in magenta. Each residue is colored by its positional median absolute binding residual. **h**, Top views of three pockets located distal to DNA, with visible residues of the pockets labeled with the color code corresponding to the structural annotation of the residue. **i**, Side view of Pocket 1, showing all its constituent residues. Source data

We next considered residues beyond the DNA-binding interface (Extended Data Fig. 4c–g). The absolute binding residuals of mutations decayed approximately exponentially with minimal three-dimensional (3D) distance from DNA (Fig. 4a). This trend is further illustrated by plotting the median absolute binding residuals per site (Fig. 4b) and visualized on the DNA-bound protein structure (Fig. 4c and Extended Data Fig. 4e,g). This distance-dependent decay of allosteric effects is strikingly similar to that observed in protein–protein interaction domains^25,26,28^, a hormone receptor^32^ and an enzyme^27^, suggesting it is likely to be a universal principle of protein biophysics.

### Allosteric anisotropy and hotspots

Although the allosteric effects of mutations on DNA binding generally decay with distance, there is a wide dispersion of binding residuals for different mutations (Fig. 4a) and residues (Fig. 4b) at each distance. To better characterize this variation in allostery across the protein, we first calculated the deviation of each site’s median absolute binding residual from the value expected based on its distance to DNA (Fig. 4d), then mapped these distance-corrected absolute binding residuals onto the structure of the protein (Fig. 4e and Extended Data Fig. 4h).

Mutational effects on binding are particularly strong in a spatially clustered set of 16 residues (median distance-corrected absolute binding residual > 0) located on the far side of Helix 3 from the DNA-binding interface (Fig. 4e and Extended Data Fig. 4h), including partially buried residues A217, Y218 and L232 and fully buried residues L214, V264 and F268. These connect surface-exposed residues F215, E261 and R278 to four DNA-contacting residues (W267, A269, R271 and E274) (Extended Data Fig. 4h).

We define allosteric hotspots as positions outside the binding interface where mutations show distance-corrected absolute binding residuals significantly greater than zero (one-sample, one-sided Wilcoxon signed-rank test, Benjamini–Hochberg (BH) false discovery rate (FDR) < 0.05). In total, HNF1A POU_H_ has seven allosteric hotspots (Fig. 4b). All these localize within the interconnected cluster of 16 residues showing higher-than-expected binding residuals (Fig. 4e and Extended Data Fig. 4h). This cluster extends from the surface-exposed residue F215 through core residues L214, A217, Y218, L232, C236 and F268, and links to two DNA-contacting residues, W267 and E274, both of which also exhibit elevated distance-corrected absolute binding residuals (Fig. 4e and Extended Data Fig. 4h).

### HNF1A POUH surface pockets

For regulation and drug discovery, solvent-accessible surface sites and pockets are of particular interest. In total, 112 mutations in 11 surface sites of HNF1A have allosteric effects (FDR < 0.05 and absolute binding-residual ≥ 0.1). Using FPocket^66^ we identified six small pockets in the HNF1A POU_H_ domain, including three distal to the DNA-binding interface (Pockets 1, 2 and 5) and three overlapping the DNA interface (Pockets 3, 4 and 6) (Fig. 4f and Extended Data Table 1).

**Extended Data Fig. 5:**
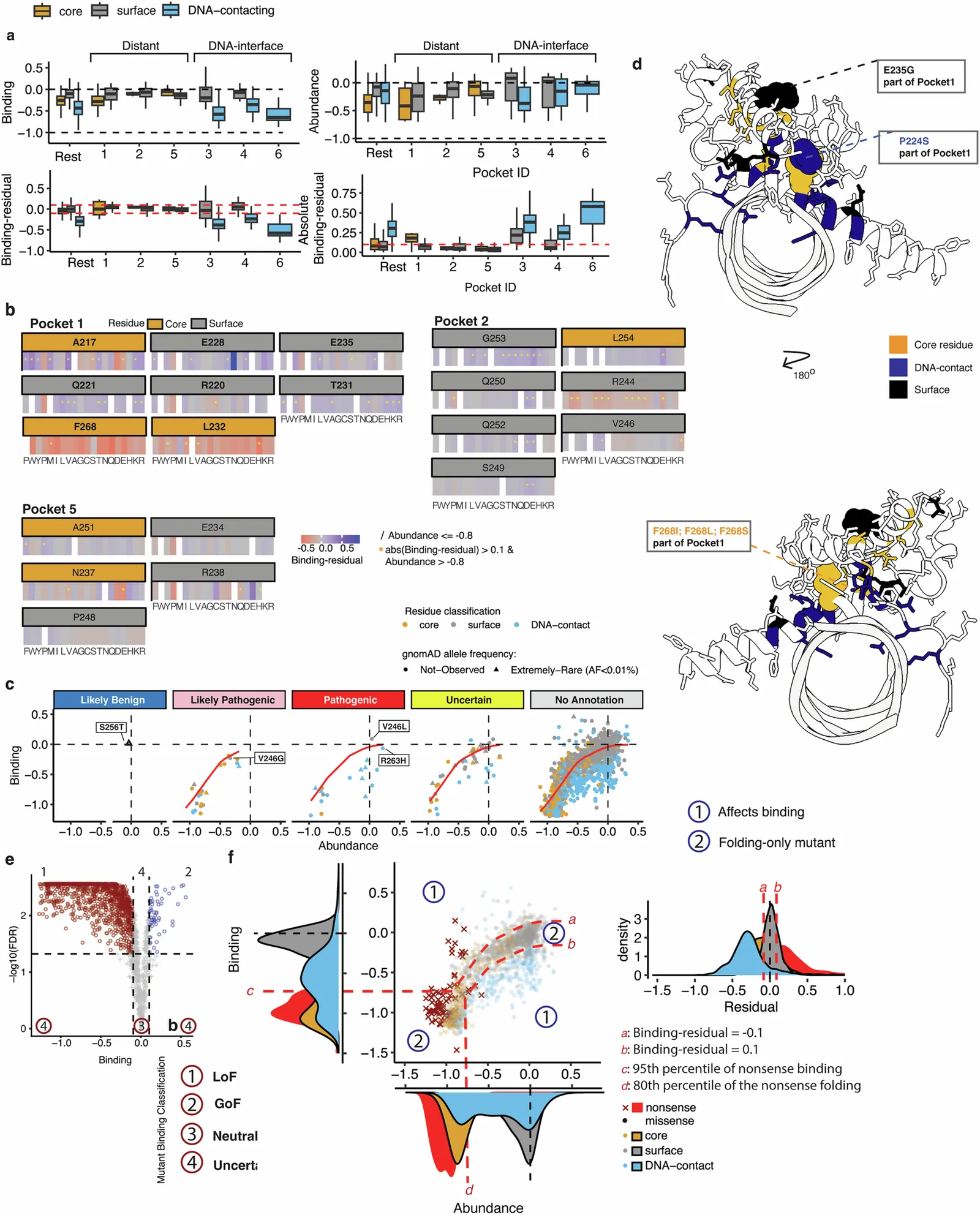
Classification of HNF1A POU_H_ variants. (**a**) Comparison of all mutational effects of residues within each pocket versus those outside the pockets of HNF1A POU_H_. The centre line indicates the median; box bounds indicate the 25th and 75th percentiles; whiskers extend from the minimum to the maximum. Red dotted lines indicate an effect size threshold of 0.1. (**b**) Binding residuals of pocket residues which do not contact DNA. (**c**) Clinical and non-annotated variants mapped in the abundance–binding phenotypic space. (**d**) Pathogenic and likely pathogenic variants mapped onto the 3D structure of the HNF1A POU_H_ bound to DNA. Colours correspond to the structural annotations. Among them, those co-localising with the prioritised druggable pockets are shown as spheres and identified with text labels. (**e, f**) Strategy for classifying variants by functional impacts, based on the volcano plot of binding effects (**e**), and by biophysical mechanism based on the abundance–binding scatter plot (**f**).

Mutations in all three binding interface pockets impair binding, but all three are very shallow pockets with low predicted druggability scores (Fig. 4f, Extended Data Fig. 5a,b and Extended Data Table 1). Of the three more distal pockets, mutations in Pocket 5 have little effect on binding. At least one substitution in each of the seven residues in Pocket 2 has a significant binding residual but the pocket is shallow and with low predicted druggability (Fig. 4h and Extended Data Fig. 5b). Mutations in all five residues in Pocket 1 affect binding, with two allosteric hotspot residues, L232 and F268 (Fig. 4b,c,e,g–i and Extended Data Fig. 4h). The pocket has a higher druggability score and mutations both enhance and impair DNA binding (Extended Data Fig. 5b). The allosteric map therefore prioritizes Pocket 1 as an allosterically active and potentially drug-sized pocket on the surface of HNF1A POU_H_ (Extended Data Table 1).

### Mechanistic classification of HNF1A POUH pathogenic variants

**Fig. 5:**
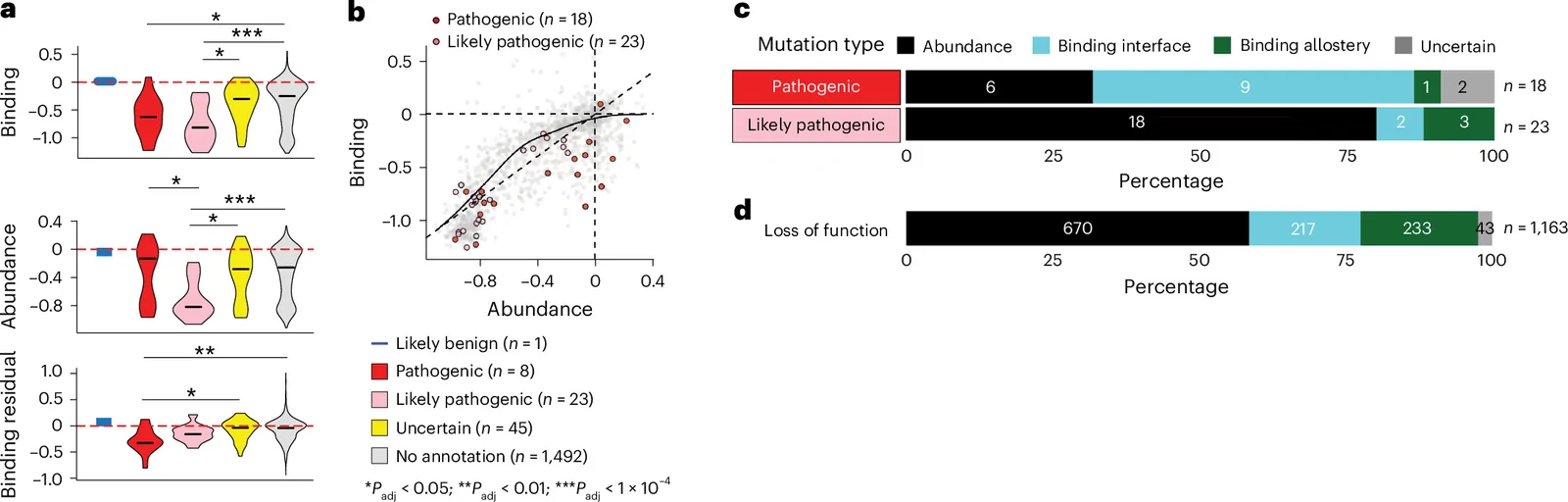
Mechanistic classification of HNF1A POU_H_ domain pathogenic variants. **a**, Functional scores compared across clinical groups. Statistical significance was assessed using Kruskal–Wallis tests followed by post hoc Dunn’s tests with Bonferroni correction; *P*_adj_ values for binding were 0.026 (pathogenic versus no annotation), 8.08 × 10^−5^ (likely pathogenic versus no annotation) and 0.021 (likely pathogenic versus uncertain). For abundance, *P*_adj_ values were 0.026 (pathogenic versus likely pathogenic), 8.33 × 10^−5^ (likely pathogenic versus no annotation) and 0.012 (likely pathogenic versus uncertain). For binding residual, *P*_adj_ values were 0.002 (pathogenic versus no annotation) and 0.015 (pathogenic versus uncertain). **b**, Two-dimensional binding-abundance landscape highlighting clinically annotated pathogenic and likely pathogenic variants. **c**,**d**, Classification of variants by biophysical category within pathogenic and likely pathogenic groups (**c**) and within loss-of-function mutations (**d**). Source data

Finally, we used the HNF1A POU_H_ TF-MAPS to understand the mechanistic effects of clinical variants (Fig. 5a). Among 18 HNF1A variants annotated as pathogenic in ClinVar, 16 reduce DNA binding (FDR < 0.05, binding < −0.1) (Fig. 5a,b and Extended Data Fig. 5c). Fourteen localize to the DNA-binding interface, while one (G207D) reduces binding by destabilizing the protein and another (E275V) acts through an allosteric mechanism (Methods, Fig. 5c and Extended Data Fig. 5c). Two variants (R263H and V246L) show no detectable effects, suggesting a different mechanism of action (Fig. 5c and Extended Data Fig. 5c). Indeed, one of these, R263H, has been shown to disrupt nuclear localization^67^.

All 23 ClinVar variants annotated as ‘likely pathogenic’ reduce DNA binding in our assay (Fig. 5a,b). Six of these are in the DNA-binding interface (Extended Data Fig. 5c); the remaining 17 act indirectly: 14 by reducing protein abundance and 3 by allosteric mechanisms (Fig. 5c and Extended Data Fig. 5c). Five likely pathogenic variants (P224S, E235G, F268I, F268L and F268S) map to residues within Pocket 1 (Extended Data Fig. 5d).

Overall we identify a total of 1,163 missense variants that significantly reduce DNA binding (Fig. 5d and Extended Data Fig. 5e). Of these, 57.6% destabilize the protein; the remainder are split almost equally between DNA-binding interface (18.7%) and allosteric mechanisms (20.0%) (Fig. 5d and Extended Data Fig. 5e).

This functional classification of variants is important for understanding disease mechanisms, guiding therapeutic development and stratifying patients in clinical trials. Notably, since only a minority of the known pathogenic variants in HNF1A reduce binding through destablization, small-molecule stabilizers^68^ may have limited therapeutic benefit for most patients.

### Functional and allosteric maps of FOXG1 and FOXP1 DBDs

**Extended Data Fig. 6:**
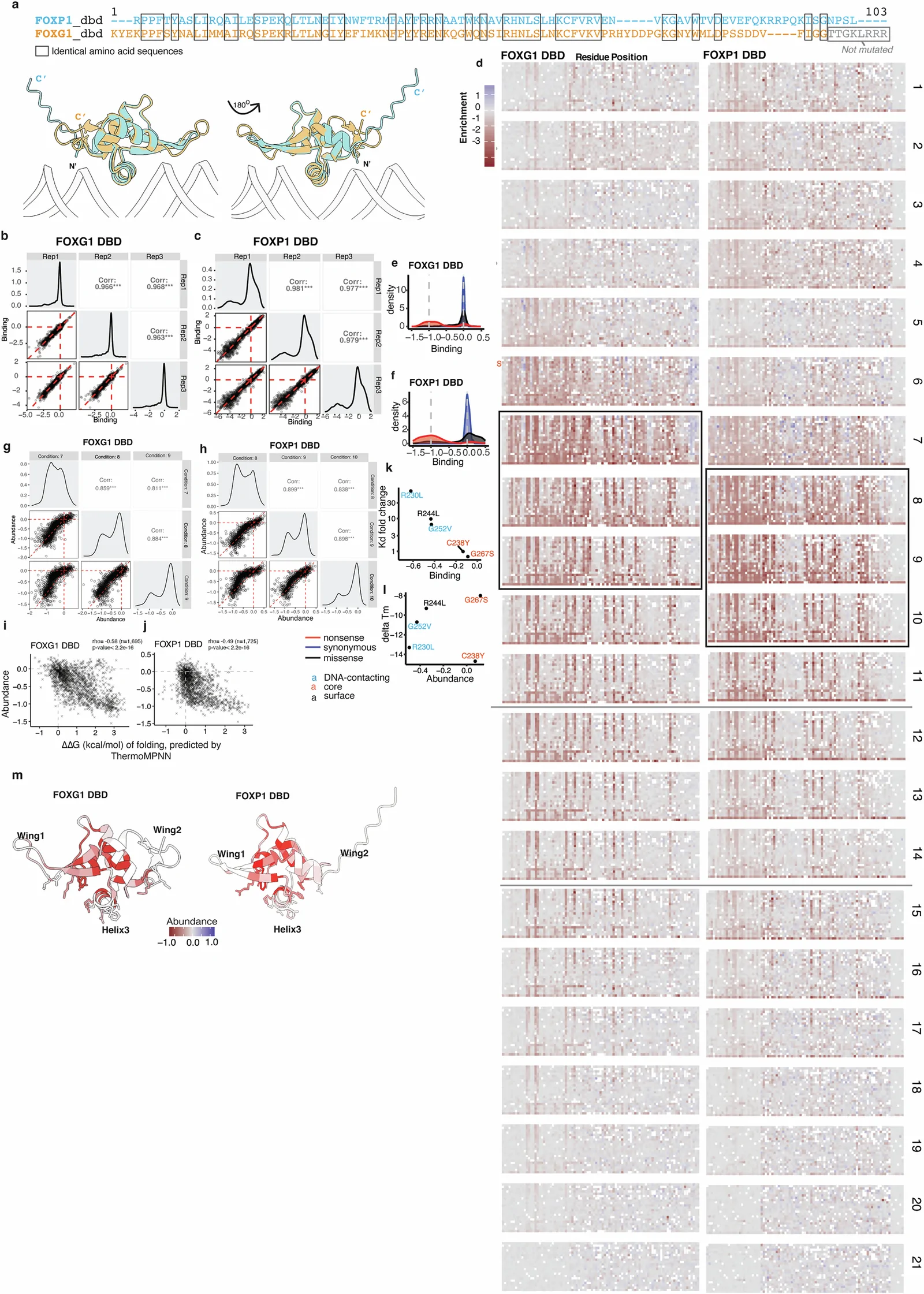
FOXG1 and FOXP1 DBDs binding and abundance measurements. (**a**) Sequence alignment of FOXG1 and FOXP1 forkhead DBDs. Grey residues in FOXG1 were not mutated in the study. (**b, c**) Correlation matrices showing binding assay reproducibility for FOXG1 (**b**) and FOXP1 (**c**) DBDs. (**d**) Heatmaps of abundance assays across all 21 tested conditions with varying concentrations of spectinomycin, where condition ‘1’ is the highest concentration and ‘21’ the lowest. Three highlighted conditions in each panel were selected for calculating abundance scores. (**e, f**) Density plots of binding scores after normalisation across three biological replicates for FOXG1 (**e**) and FOXP1(**f**). (**g, h**) Correlation matrices showing abundance assay reproducibility for FOXG1 (**g**) and FOXP1 (**h**) DBDs at selected conditions. (**i, j**) Comparison of STS assay results with ThermoMPNN-predicted protein stability changes for FOXG1 (**i**) and FOXP1 (**j**). Statistical significance was determined using a two-sided Spearman’s rank correlation coefficient test with a large-sample normal approximation. No adjustments were made for multiple comparisons. (**k, l**) Correlation between published Kd values and FOXG1 binding scores (**k**), Tm changes and abundance scores (**l**). Each mutation is coloured by the residue structural annotation. (**m**) Median mutational effects per amino acid residue for FOXG1 and FOXP1 DBD abundance. Source data

We next applied TF-MAPS to two forkhead TFs, FOXG1 and FOXP1, from a different DBD structural family, to compare the functional and allosteric maps of two homologous DBDs. Both proteins bind the canonical forkhead motif RYAAAYA^48,69–72^ (R = G/A, Y = C/T) through Helix 3 of the winged helix-turn-helix domain. Structural alignment of DNA-bound FOXG1 and FOXP1 DBD monomers shows high structural conservation (root mean square deviation = 0.61 Å; Extended Data Fig. 6a). The sequences share 50% identity and 69% similarity with 6% gaps (Extended Data Fig.6a).

**Fig. 6:**
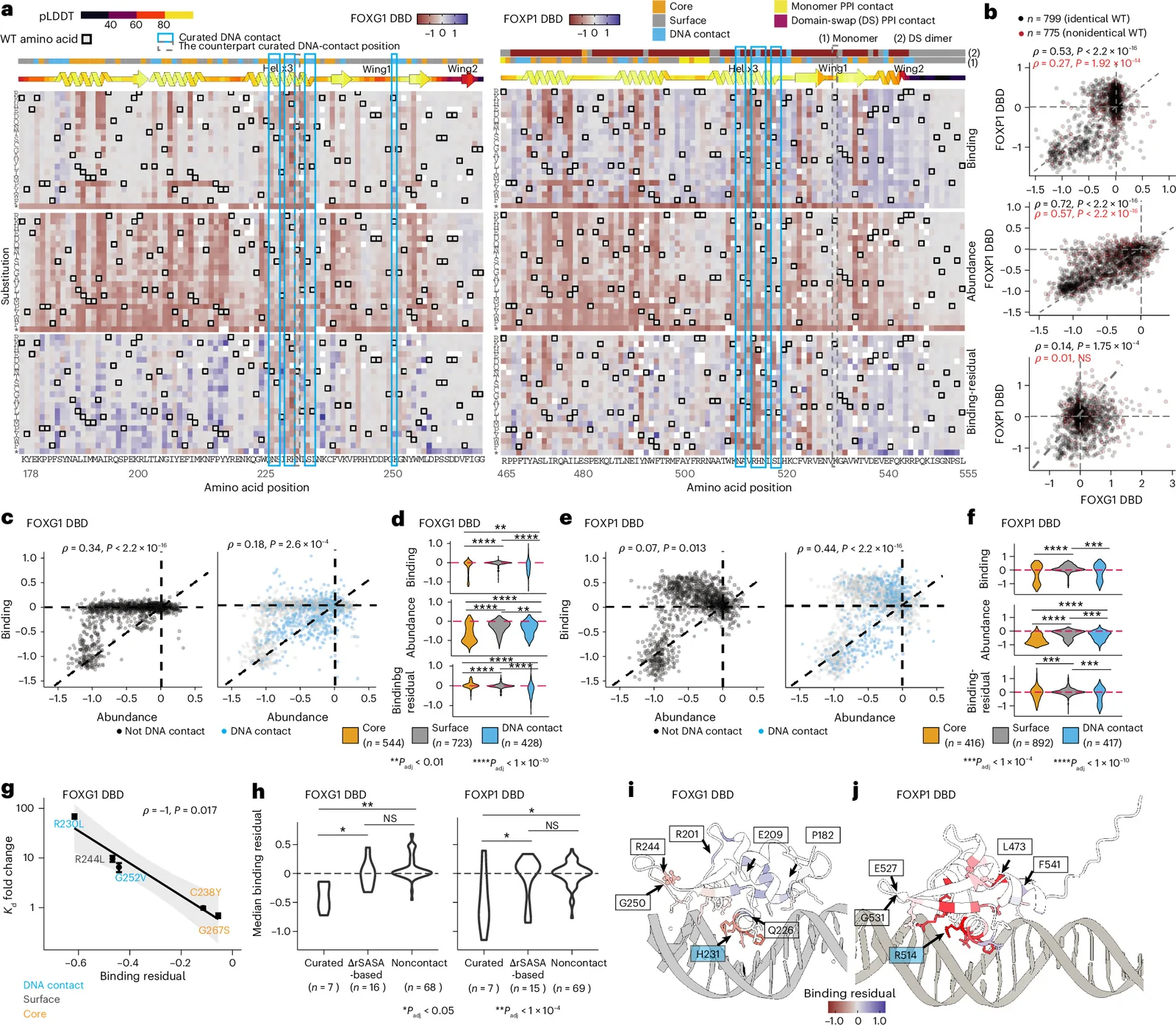
TF-MAPS for FOXG1 and FOXP1 DBDs. **a**, Heatmaps showing mutational effects with missing values left blank. **b**, Correlations between matching variants’ binding, abundance and binding residuals (from top to bottom) between FOXG1 and FOXP1 DBDs. **c**,**e**, Relationship between abundance and binding effects for FOXG1 (**c**) and FOXP1 (**e**) DBDs. **d**,**f**, Comparison of mutational effects across all structural classes for FOXG1 (**d**) and FOXP1 (**f**) DBDs. *P* values are shown only for statistically significant comparisons. *P*_adj_ values for FOXG1 DBD, comparing core and DNA-binding residues, core and surface residues, and surface and DNA-binding residues, are as follows: for binding, 0.0027, 3.84 × 10^−17^ and 5.28 × 10^−3^; for abundance, 1.54 × 10^−19^, 3.3 × 10^−42^ and 0.0071; and for binding residual, 8.99 × 10^−54^, 4.77 × 10^−20^ and 1.88 × 10^−14^ (**d**). For FOXP1 DBD, the corresponding *P*_adj_ values are: for binding, 0.13, 7.33 × 10^−13^ and 2.69 × 10^−6^; for abundance, 1.88 × 10^−24^, 7.72 × 10^−66^ and 9.92 × 10^−7^; and for binding residual, 1.00, 1.38 × 10^−5^ and 1.74 × 10^−6^ (**f**). **g**, Correlation between the published *K*_d_ values and the binding residual of FOXG1. Data are presented as the mean with a 95% confidence interval (CI). The shaded area surrounding the linear regression line indicates the 95% CI for the regression line. **h**, Comparison of mutational effects across all structural classes for FOXG1 and FOXP1. *P*_adj_ values for FOXG1 DBD are 0.02 (curated and ∆rSASA-based contacts), 5.5 × 10^−5^ (curated contacts and noncontact residues) and 0.22 (∆rSASA-based contacts and noncontact residues). For FOXP1 DBD, the corresponding *P*_adj_ values are 0.036 (curated and ∆rSASA-based contacts), 0.039 (curated contacts and noncontact residues) and 1.0 (∆rSASA-based contacts and noncontact residues). **i**,**j**, FOXG1 (**i**) and FOXP1 (**j**) DBD binding residuals. White-boxed positions indicate residues within Wing 1 that show large absolute binding residuals but do not contact DNA. For comparison, the DNA-contacting residue with the largest binding residual is highlighted with a colored box. Statistical significance in **b**, **c**, **e** and **g** was determined using a two-sided Spearman’s rank correlation coefficient test with a large-sample normal approximation. No adjustments were made for multiple comparisons. Statistical significance in **d**, **f** and **h** was assessed using Kruskal–Wallis tests followed by post hoc Dunn’s tests with Bonferroni correction. Source data

**Extended Data Fig. 7:**
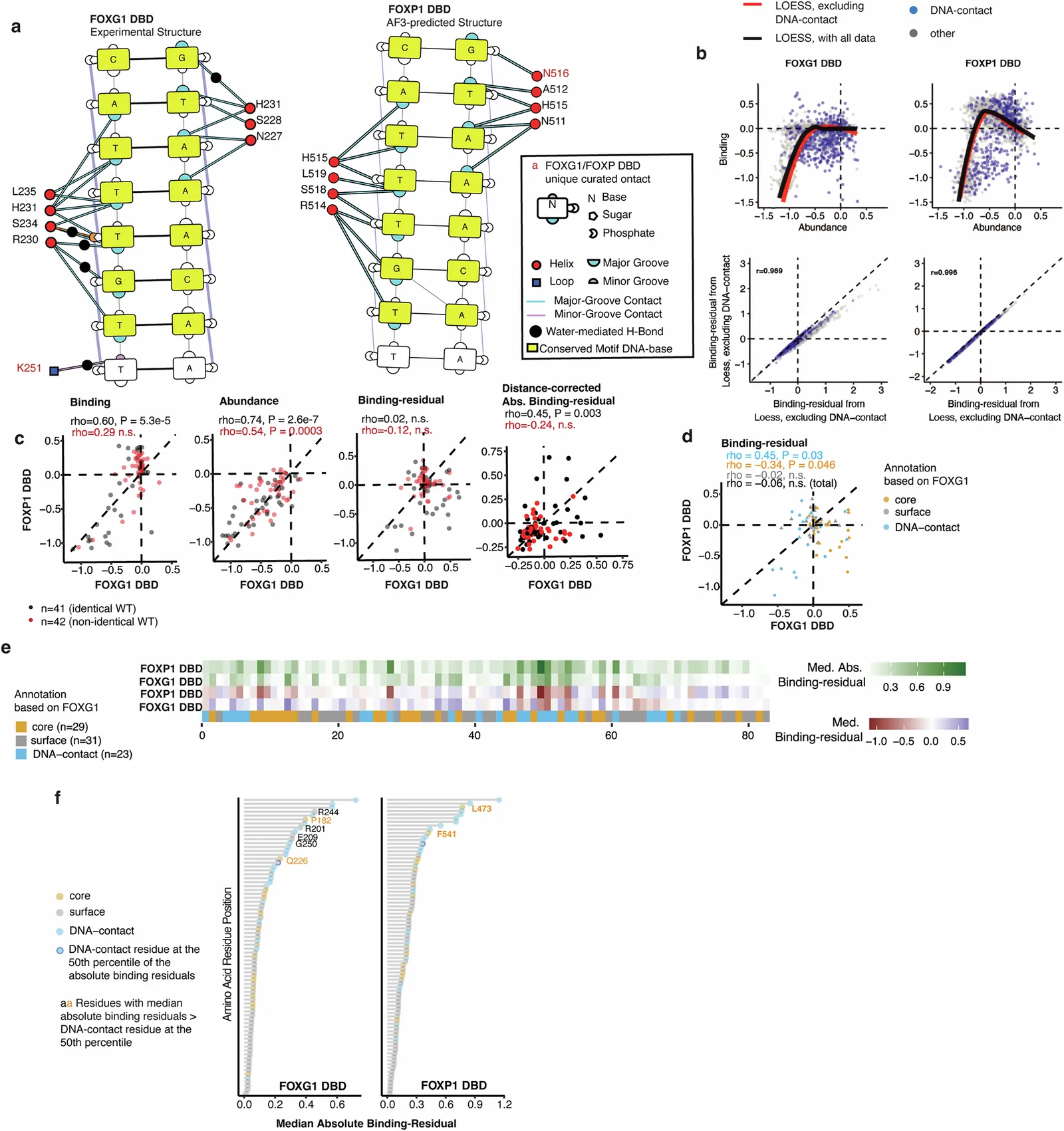
TF-MAPS for FOXG1 and FOXP1 DBDs. (**a**) DNA-contact map generated with DNAproDB based on the DNA-bound structure of FOXG1 (PDB:7CBY) and AlphaFold3-predicted structure of the DNA-bound FOXP1 monomer DBDs. (**b**) Comparisons of the LOESS fit (upper row) and binding residuals (bottom row) between conditions with and without DNA-contacting residues, for FOXG1 (left) and FOXP1 (right) DBDs. (**c, d**) Correlations between median binding residuals of matched positions in FOXG1 and FOXP1 DBDs coloured by identical and non-identical WT sequences (**c**) and structural annotation (**d**). Statistical significance was determined using a two-sided Spearman’s rank correlation coefficient test with a large-sample normal approximation. No adjustments were made for multiple comparisons. (**e**) Heatmaps of aligned positional median binding-residuals and absolute binding-residuals for FOXG1 and FOXP1 DBDs. Positions are re-indexed based on sequence alignment. (**f**) Ordered positional median absolute binding residuals. Source data

Mutational effects were highly reproducible across biological replicates in the B1H assay (median Spearman’s *ρ* = 0.966 for FOXG1 DBD, 0.979 for FOXP1 DBD) and across three consecutive drug concentrations in the STS assay (median Spearman’s *ρ* = 0.859 for FOXG1 DBD, 0.898 for FOXP1 DBD) (Extended Data Fig. 6b–h). Mutational effects on abundance are well correlated between the two proteins (Spearman’s *ρ* = 0.72 for aligned identical wild-type (WT) residues and 0.57 for different WT residues) but less so than replicate measurements for each protein alone (Fig. 6a,b and Extended Data Figs. 6m and 7c). Mutational effects on binding are less strongly correlated (Spearman’s *ρ* = 0.53 for aligned identical WT residues and 0.27 for different WT residues) (Fig. 6b and Extended Data Figs. 6b,c,e,f and 7c).

As for HNF1A POU_H_, many changes in binding can be accounted for by changes in abundance (Fig. 6c–f). Fitting a LOESS curve to the binding-abundance measurements yields a binding residual per mutation (last row in Fig. 6a and Extended Data Fig. 7b), which correlates very well with independently measured in vitro dissociation constants (*K*_d_) for FOXG1^72^ (Spearman’s *ρ* = −1) (Fig. 6g and Extended Data Fig. 6k). For both proteins, the binding residuals are largest at the interface, especially in Helix 3 (Fig. 6a,i,j and Extended Data Fig. 7a). Across two proteins, binding residuals correlate weakly but significantly for identical residues (Spearman’s *ρ* = 0.14), but there is no correlation for nonidentical residues (Fig. 6b and Extended Data Fig.7e,f).

Mutations in curated DNA contacts identified by DNAproDB^59^ strongly affect binding, unlike in other ∆rSASA-based DNA contacts (Fig. 6h). This contrasts with HNF1A, where no difference is observed between curated and the rest of the DNA contacts in their effects on DNA binding (Fig. 3d), indicating that in FOXG1 and FOXP1 only key interacting residues mediate DNA binding.

We observe strong binding residuals within Wing 1 of FOXG1 and FOXP1 DBDs despite no direct DNA contacts in either experimental or predicted structures (Fig. 6i,j). Mutations with large binding residuals include those at an aligned glycine (FOXG1 G250, FOXP1 G531; medians −0.31, 0.22) and nearby charged residues (FOXG1 R244 and an oppositely charged FOXP1 E527; medians −0.46, −0.26) (Fig. 6i,j and Extended Data Fig. 7a). Although these charged side chains do not directly contact DNA in the experimental structures of FOXG1 and FOXP1^72–74^, the Wing 1 region of another forkhead FOXD3 DBD has been suggested to form dynamic interactions with DNA^75^. Our data suggest a similar mechanism in FOXG1 and FOXP1 DBDs or an allosteric mechanism.

Unlike the HNF1A POU_H_ domain where interface binding residuals dominate (Extended Data Fig.4f), six surface and core residues in FOXG1 DBD (R244, P182, R201, E209, G250, Q226) and two core residues in FOXP1 (L473 and F541) show median absolute binding residuals exceeding half those of DNA‑contacting residues (Extended Data Fig. 7f). R244 and G250 (FOXG1) and L473 (FOXP1) have negative median binding residuals, whereas the remaining four FOXG1 residues and FOXP1 L473 show positive median binding residuals. None of these residues are aligned positions between the two DBDs; the six FOXG1 residues are spatially dispersed (Fig. 6i) whereas the two FOXP1 core residues (L473 and F541) lie in the two closelypacked helices (Fig. 6j).

### Distance-dependent allosteric decay and anisotropy

**Fig. 7:**
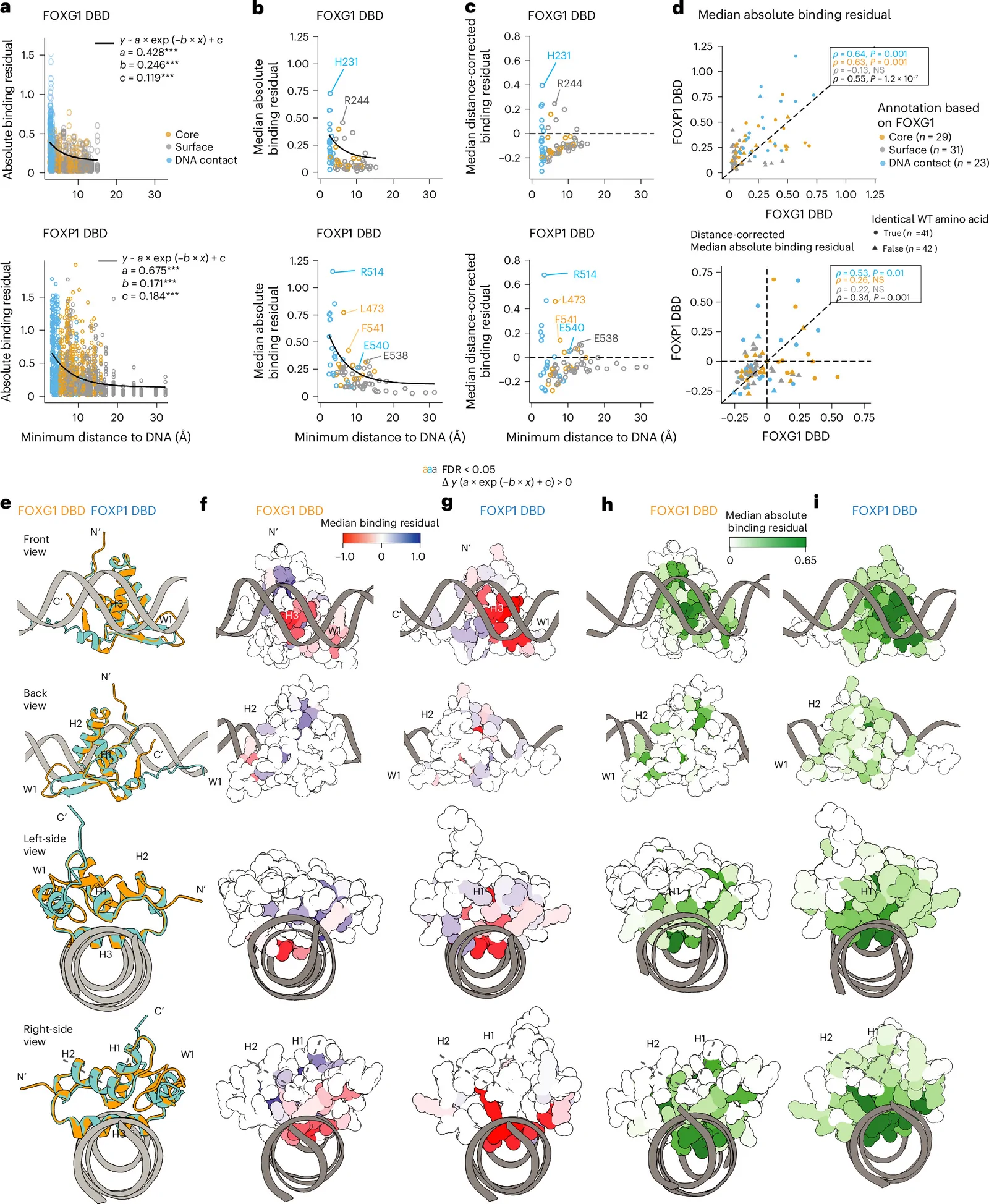
Binding interface and allosteric decay for FOXG1 and FOXP1 DBDs. **a**,**b**, Distance-dependent decay plots of absolute binding residuals for all mutations (**a**) and for position-wise median (**b**). **c**, Distance-corrected binding residuals after fitting a decay curve based on distance to DNA, highlighting positions with significantly elevated residuals compared to decay-predicted values (FDR < 0.05, one-sample Wilcoxon signed-rank tests followed by BH correction). **d**, Correlations between matching positions’ median absolute binding residual and distance-corrected absolute binding-residual scores between FOXG1 and FOXP1 DBDs. Statistical significance was determined using a two-sided Spearman’s rank correlation coefficient test with a large-sample normal approximation. No adjustments were made for multiple comparisons. **e**–**i**, Structural views of aligned FOXG1 and FOXP1 DBDs (**e**); median binding residuals of FOXG1 (**f**) and FOXP1 (**g**) DBDs; and median absolute binding-residuals of FOXG1 (**h**) and FOXP1 (**i**) DBDs from several perspectives. H, helix; W, wing. Source data

Absolute binding residuals for FOXG1 and FOXP1 also decay with spatial distance to the DNA, consistent with the pattern observed in HNF1A (Figs. 4a,b and 7a). Besides this distance-dependent decay, both FOXG1 and FOXP1 exhibit notable dispersion of absolute binding residuals at each distance (Fig. 7a–c), indicative of functional and allosteric anisotropy where mutations in certain residues exert disproportionately strong effects, as observed in HNF1A.

Distance-corrected absolute binding residuals (dispersion) are correlated between the two proteins (Spearman’s *ρ* = 0.34) (Fig. 7d and Extended Data Fig. 7c), driven by mutations in DNA-contacting residues (Spearman’s *ρ* = 0.53) and identical noncontacting amino acid residues (Spearman’s *ρ* = 0.45), with no significant correlation for nonidentical sites (Extended Data Fig. 7c–e).

Significantly elevated distance‑corrected median absolute binding residuals identified two FOXG1 positions (DNA-contacting H231; surface R244) and five FOXP1 positions (DNA-contacting R514, E540; core L473, F541; surface E538) (Fig. 7b). None of these are aligned positions in FOXG1 and FOXP1. Allosteric anisotropy between the two domains is thus partially conserved, with differences concentrated at nonconserved sites.

### FOXG1 and FOXP1 DBDs have diverged allosteric surfaces

We next compared the effects of surface mutations in the two homologous DBDs, visualized across four orientations relative to the DNA interface (Fig. 7e–i). In the front view, mutations in Helix 3 and Wing 1 show strong negative binding residuals in both proteins (top row, Fig. 7e–g), whereas N- and C-terminal regions facing the DNA backbone show positive residuals for both DBDs (Fig. 7f,g).

From the back view, mutational effects are more divergent. FOXG1 DBD exhibits stronger and more concentrated allosteric mutational effects, particularly along the left side of the protein surface. In comparison, FOXP1 DBD shows milder and more diffusely distributed effects (second row, Fig. 7e–g).

From the left-side view, the C-terminal end of Helix 3 shows much stronger binding residuals in FOXP1 compared to the corresponding positions in FOXG1 (third row, Fig. 7e–g). On the right-side view, the Helix 1–Helix 2 region shows the greatest differences: several residues in FOXG1 have positive median binding residuals, whereas FOXP1 counterparts are near zero. However, FOXP1’s absolute binding residuals are markedly higher in this region, reflecting mixed positive and negative binding-residual effects that cancel out (bottom row, Fig. 7e–i).

Overall, FOXP1 DBD shows more pronounced allosteric effects, reflected by larger absolute binding residuals and the slower distant-dependent decay (decay rate: 0.246 for FOXG1 DBD and 0.171 for FOXP1 DBD) (Fig. 7a,h,i). Surface binding residuals are not correlated in the two proteins (Fig. 7d and Extended Data Fig. 7d), unlike core (Spearman’s *ρ* = -0.34; absolute residuals Spearman’s *ρ* = 0.63) and DNA-contacting residues (Spearman’s *ρ* = 0.45; absolute residuals Spearman’s *ρ* = 0.64) (Fig. 7d and Extended Data Fig. 7d,e). This surface-specific divergence in allostery in structurally homologous proteins has also been observed in PDZ domains^28^.

### FOXG1 and FOXP1 DBD pockets

**Fig. 8:**
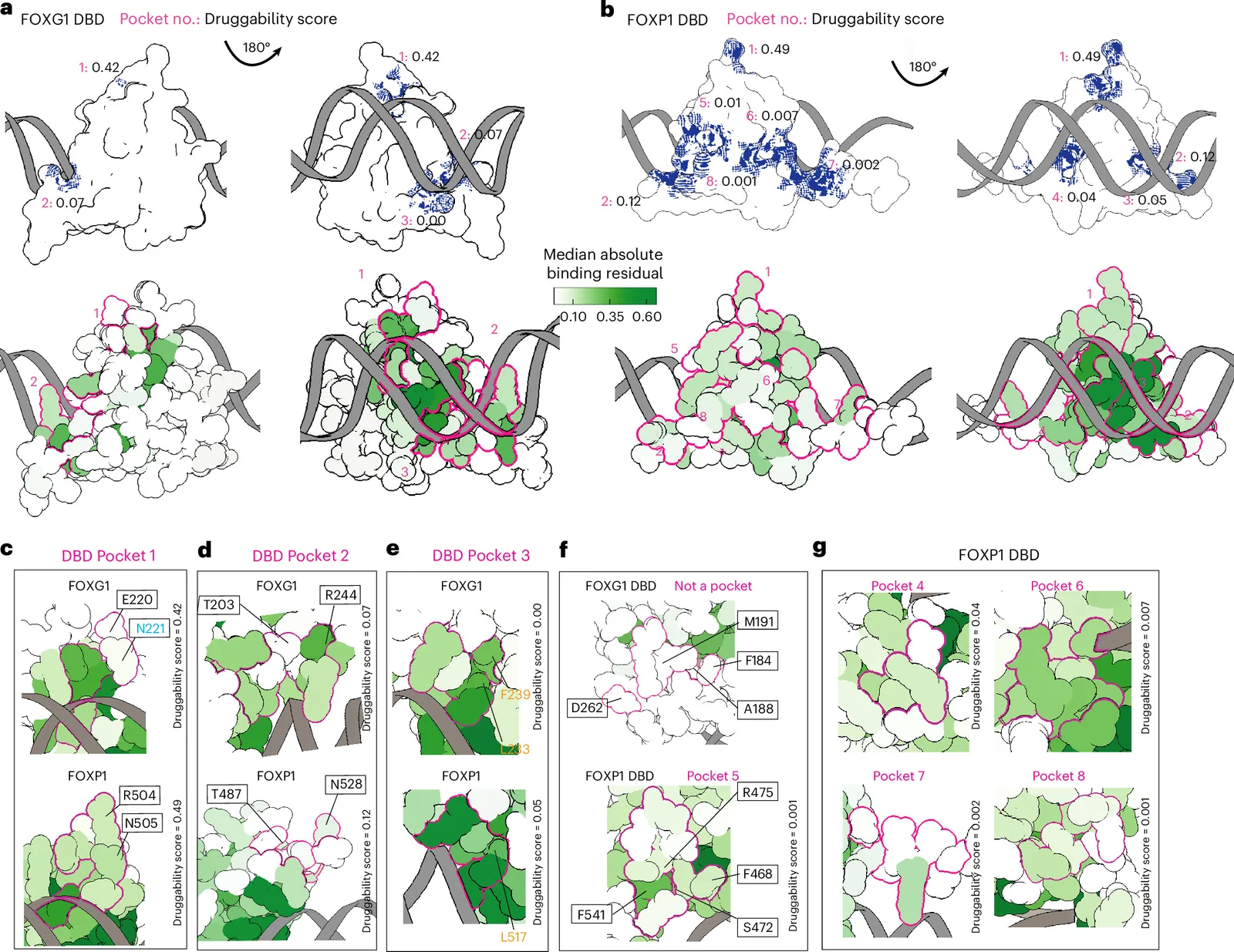
Allosteric surfaces and pockets of FOXG1 and FOXP1 DBDs. **a**,**b**, All pockets identified by the FPocket algorithm shown on the DNA-bound structure for FOXG1 (**a**) and FOXP1 (**b**) DBDs; pocket-forming residues are shaded. **c**–**g**, Pocket-centric view, with each structurally aligned pocket pair: FOXG1 and FOXP1 DBD Pocket 1 (**c**), FOXG1 and FOXP1 DBD Pocket 2 (**d**), FOXG1 and FOXP1 DBD Pocket 3 (**e**) and FOXP1 DBD Pocket 5 with the corresponding positions in FOXG1 DBD (**f**). The unmatched pockets of FOXP1 DBD are shown together (**g**). Pockets are marked in magenta and individual residues are colored by their positional median absolute DNA-binding residuals. Source data

**Extended Data Fig. 8:**
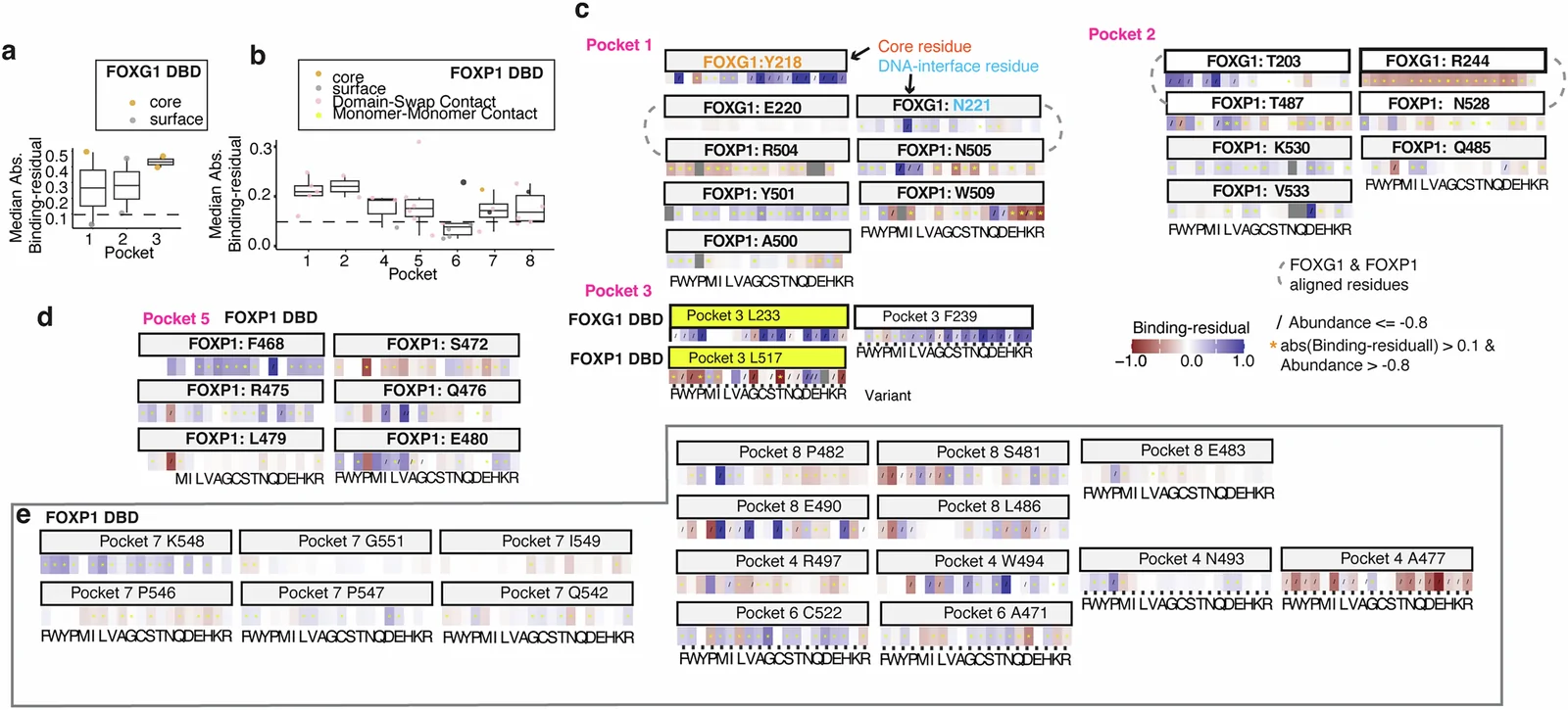
TF-MAPS positional median comparisons for FOXG1 and FOXP1 DBDs. (**a**, **b**) Median absolute binding-residuals of each residue within each pocket of FOXG1 (**a**) and FOXP1 (**b**). The centre line indicates the median; box bounds indicate the 25th and 75th percentiles; whiskers extend from the minimum to the maximum. Only residues outside the DNA interfaces with the median abundance score > -0.8 are shown as overlaid individual datapoints. Pocket 3 of FOXP1 is not shown because the only non-DNA contacting residue L517 is highly destabilising with the median abundance score < -0.8. (**c**) Binding-residuals of aligned pockets of FOXG1 and FOXP1. DNA-contacting positions are excluded except for a DNA-contacting FOXG1 residue N221 of Pocket 1, to compare with its aligned non-DNA-contacting FOXP1 residue N505. (**d, e**) Binding residuals of Pocket 5 of FOXP1 (**d**) and other FOXP1 pockets (**e**).

Using FPocket^66^, we identified three structural pockets in FOXG1 and eight in FOXP1 DBDs (Methods, Fig. 8a,b and Extended Data Table 1). All three FOXG1 DBD pockets are located near the DNA and are structurally conserved in FOXP1. FOXP1 DBD also has an additional five distal pockets not detected in FOXG1 (Fig. 8a,b and Extended Data Fig. 8a,b). Most pockets are shallow and have low predicted druggability (Fig.8a,b and Extended Data Table 1), with Pocket 1 ranking highest in druggability in both proteins. Pocket 1 is located in the binding interface. Many mutations in this pocket strongly impair DNA binding, identifying it as a potentially druggable hotspot required for DNA binding (Fig. 8a–e and Extended Data Fig. 8a–c).

Mutations in the distal pockets of FOXP1 DBD strongly affect DNA binding, suggesting the pockets are allosterically active (Fig. 8b,f,g and Extended Data Fig. 8a,d,e). Mutations in Pocket 6 have particularly strong allosteric effects (Fig. 8g and Extended Data Fig. 8e). However, targeting these shallow pockets is probably challenging because they have low predicted druggability (Fig. 8b and Extended Data Table 1).

In contrast to HNF1A POU_H_, the functional maps therefore identify a binding interface hotspot pocket in FOXG1 and FOXP1 DBDs and prioritize it as the top pocket to target therapeutically with small molecules to inhibit binding.

### Mechanistic classification of FOXG1 and FOXP1 pathogenic variants

**Extended Data Fig. 9:**
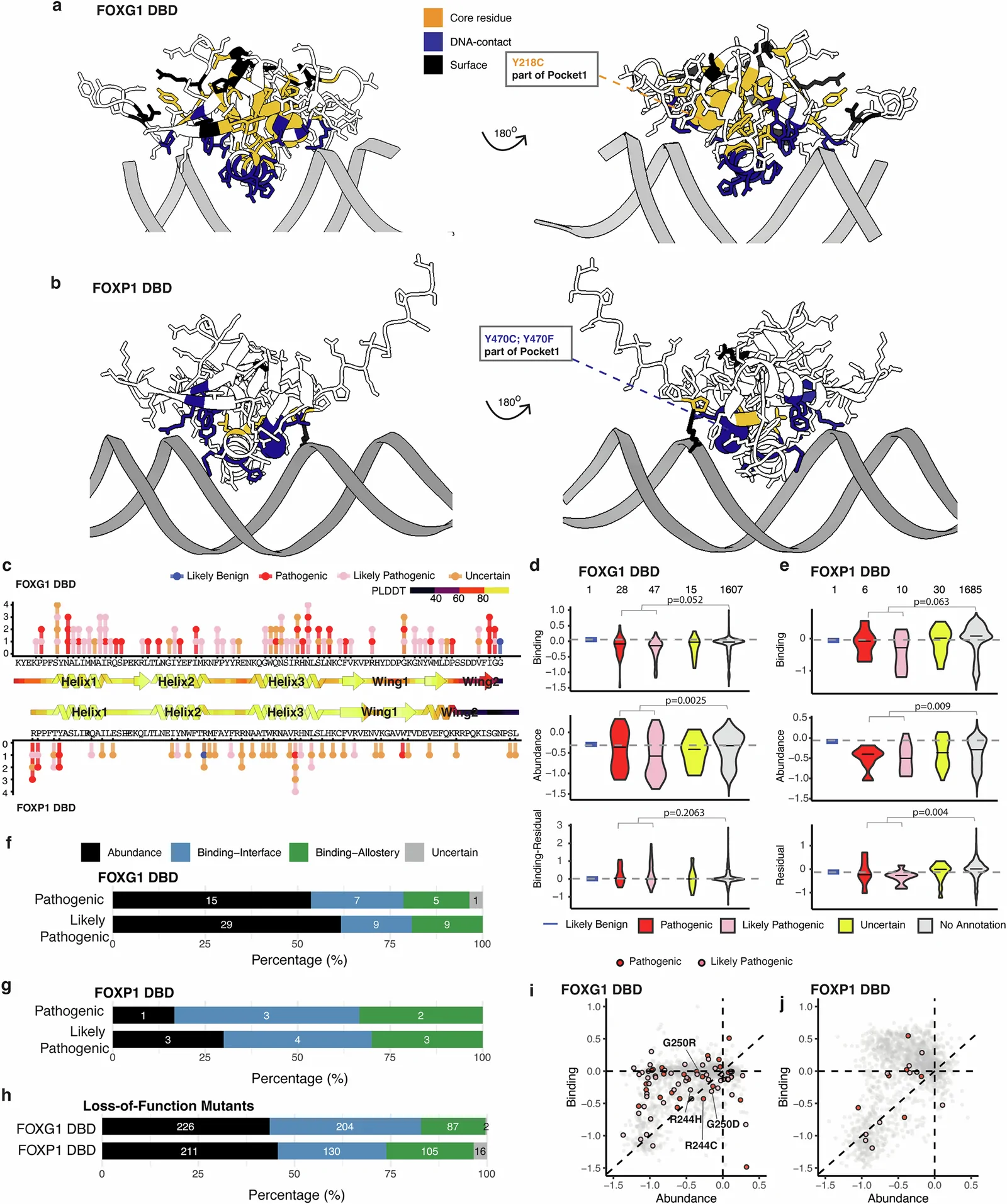
Mechanistic classification of FOXG1 and FOXP1 DBD pathogenic variants. (**a, b**) Pathogenic and likely pathogenic variants are mapped onto the DNA-bound 3D structures of the FOXG1 DBD (**a**) and FOXP1 DBD (**b**), with colours corresponding to the structural annotations. Among them, those colocalising with the prioritised druggable pockets (Pocket 1) are shown as spheres and labelled. (**c**) Clinically annotated variants are marked linearly along the aligned FOXG1 and FOXP1 DBDs. (**d, e**) Functional scores compared across clinical groups for FOXG1 (**d**) and FOXP1 (**e**) DBDs. Kruskal–Wallis shows no statistical significance (all P > 0.05). Annotated p-values are from Wilcoxon rank-sum tests comparing the combined pathogenic/likely pathogenic group with the non-annotated group. (**f-h**) Classification of variants by biophysical category within pathogenic and likely pathogenic groups for FOXG1 (**f**) and FOXP1 (**g**) DBDs; and within loss-of-function mutations (**h**). (**i, j**) Two-dimensional binding-abundance landscape highlighting clinically annotated pathogenic and likely pathogenic variants in FOXG1 (**i**) and FOXP1 (**j**) DBDs. Source data

In FOXG1 DBD, 28 pathogenic and 47 likely pathogenic variants span 83% of the core residues (24 of 29) and 70% of the DNA-contacting residues (16 of 23) (Extended Data Fig. 9a,c). Several pathogenic and likely pathogenic mutations at surface positions, R244, G250 and R201, show large binding residuals (Fig. 6i and Extended Data Fig. 9a,i). A likey pathogenic variant Y218C localizes to the prioritized druggable Pocket 1 (Extended Data Fig. 9a).

In the FOXP1 DBD, there are fewer annotated pathogenic (*n* = 6) and likely pathogenic variants (*n* = 10) (Extended Data Fig. 9b,j). Over half (9 of 16) affect DNA contacts, 2 lie in the monomer core, and 4 surface pathogenic or likely pathogenic mutations map to the monomer–monomer interface (R465) or the domain-swapped interface (Q476, L517, F502), important for bridging two bound DNA sequences for long-range chromatin interactions^76,77^. The DNA-binding residue Y470 lies in FOXP1’s prioritized Pocket 1 (Extended Data Fig. 9b). Both proteins also harbor small clusters of pathogenic and likely pathogenic variants in the N‑terminal loop, Helix 1 head and conserved arginine (FOXG1 R230, FOXP1 R514) helix (Extended Data Fig. 9c).

For FOXG1 DBD pathogenic (*n* = 28) and likely pathogenic (*n* = 47) variants, 58.6% (53.6% for pathogenic and 61.7% for likely pathogenic) affect protein abundance, 21.3% (25% and 19%, respectively) affect binding at the DNA‑binding interface and 18.6% (17.8% and 19.1% respectively) affect binding allosterically (Extended Data Fig. 9f).

For FOXP1 DBD pathogenic (*n* = 6) and likely pathogenic (*n* = 10) variants, 30.8% (one of pathogenic and three of likely pathogenic) affect abundance, 53.8% (three and four, respectively) map to the binding interface and affect binding, and 38.5% (two and three, respectively) have an allosteric mechanism (Extended Data Fig. 9g).

The FOXG1 and FOXP1 DBDs show similar overall proportions of abundance-driven loss of function (43.5% and 45.6%, respectively; Extended Data Fig. 9h). Among the remaining loss-of-function variants, FOXG1 exhibits a higher proportion of loss-of-function variants in the binding interface (39.3% versus 28.1% for FOXP1), whereas FOXP1 shows a higher proportion of allosteric loss-of-function (22.7% versus 16.8% for FOXG1) (Extended Data Fig. 9h), consistent with the more prevalent allostery in FOXP1 DBD (Fig. 7).

## Discussion

Here we present TF-MAPS—a general and fast platform for generating complete functional and allosteric maps for DNA-binding proteins. TF-MAPS allows identification of energetically important residues in binding interfaces, identifies variant effect mechanisms and produces global maps of allosteric regulation in DNA-binding proteins. The platform is highly scalable: bacterial selection enables straightforward cloning and rapid small-volume screening.

Functional maps of HNF1A, FOXG1 and FOXP1 DBDs provide important insights: (1) all three are allosterically active and show distance-dependent allosteric decay—a pattern observed in protein interaction domains^25,26,28^ and an enzyme^27^, suggesting a general principle of protein biophysics; (2) allostery is anisotropic, with a set of allosteric hotspot residues with unusually strong effects on binding when mutated; (3) FOXG1 and FOXP1’s allosteric surfaces are divergent, indicating that TF family members may have unique targetable allosteric sites^28^; (4) TF-MAPS identify key DNA-contacting residues and provide experimental support for an extended DNA-binding interface for HNF1A POU_H_; (5) TF-MAPS provide reference atlases for clinical genetics, classifying the mechanisms-of-action of pathogenic variants—approximately half (44–58%) of loss-of-function variants reduce protein abundance, between 19% and 39% are located in the binding interface, and the rest have an allosteric mechanism; (6) TF-MAPS identify functionally important surface pockets (in FOXG1 and FOXP1, a potentially druggable DNA-interface pocket is most promising, whereas in HNF1A, a distal allosteric pocket shows large effects on binding); and (7) mutations in these pockets can reduce or increase DNA binding (Extended Data Figs. 5b and 8), indicating that small-molecule binding could be used to inhibit or enhance activity. For TFs with mutations within these pockets, mutation-specific small molecules may be necessary to modulate the effects.

The current TF-MAPS approach has several limitations—primarily that measurements are performed in bacteria on DNA that lacks the human chromatin context. Future work will be needed to assess how chromatinization influences mutational effects and allosteric behavior^78,79^. Additional mechanisms of pathogenicity exist, including changes in protein localization^61,80–82^, post-translational modifications^83^ and alternative splicing^84^, that will not be captured by our platform. In addition, ligand-specific allostery^26^ or ligand-binding specificity regulated by mutations allosterically^85,86^ are not captured. It will be interesting to quantify the extent to which allosteric effects are DNA-sequence-specific for TFs. In future work, it will also be important to extend functional and allosteric mapping to non-DBDs and the often large intrinsically disordered regions of human TFs^87,88^.

A key strength of the TF-MAPS approach is its speed, generalizability and scalability. With binding preferences available for hundreds of human TFs^4^, we believe functional and allosteric mapping can be extended to a very large number of TFs relevant to both medicine and biotechnology.

Structure-based approaches identify pockets in proteins, but not the functional consequences of targeting them. TF-MAPS thus complements structural biology to functionally annotate protein structures. Contrary to the view that TFs are undruggable proteins, all three TFs studied here have (small) drug-sized pockets where mutations strongly affect DNA binding. The TF-MAPS approach is general and requires no specialist equipment. It can therefore be applied by many groups to hundreds of different TFs implicated in human disease and important for biotechnology. Functional and allosteric maps of hundreds of human TFs and other DNA-binding proteins will provide a rich resource to understand, predict and engineer DNA recognition and TF regulation. The maps will also provide mechanistic atlases of variant effects to interpret germline and somatic clinical variants. Most importantly, we believe the approach will allow the identification of pockets and surface sites where perturbations have large effects on DNA binding. If these hotspot pockets are more widespread on TFs than is currently appreciated as our data suggest, it may be possible to transform TFs into successfully drugged targets, as has been achieved for other ‘undruggable’ protein families^89^.

## Methods

### Experimental construct design

For the B1H system, the protein of interest (POI) is linked to the C terminus of the RNA polymerase α‑subunit, whereas in the STS assay the POI is expressed between two fragments of the *aadA* gene using a linker. In other words, the POI C terminus is exposed in the DNA-binding assay (B1H) but not in the abundance assay (STS). We extended the C‑terminal boundary of each construct beyond the predicted structured domain to include a short unstructured region, based on the predicted protein structure: HNF1A amino acids 197–288 (Uniprot ID P20823); FOXG1 amino acids 178–276 (Uniprot ID P55316) and FOXP1 amino acids 465–555 (Uniprot ID Q9H334). The selected binding motif sequences are ‘AGTTAATTATTAACCAA’ (CISBP-ID M10693) for HNF1A, and ‘GTAAACAA’ for FOXG1 (CISBP-ID M01976) and FOXP1 (CISBP-ID M00837, with an additional ‘T’ preceding the motif: ‘TGTAAACAA’). All the oligonucleotides used in the study are listed in Supplementary Table 1.

### Variant library design and preparation

All codon-randomized oligo pools (NNK, NNS or NNH) were ordered from Integrated DNA Technologies to substitute each codon with variants encoding 19 alternative amino acids and stop codons (Supplementary Table 1). Here, ‘N’ represents all four possible nucleotide bases. Depending on the WT nucleotide at the wobble position, we used different degenerate bases to reduce the occurrence of WT sequences and minimize the number of stop codons at each codon position. Specifically, we generally used the degenerate base K (G/T) to reduce stop codon frequency. When the wobble base is T, we used S (C/G) for substitution, and when the WT wobble base is G, we used H (A/C/T) to diversify the codon pool to minimize the occurrence of the WT nucleotide sequences.

For the 3′ primer oligonucleotide pools, the degenerate codon mixes were embedded in the reverse complement of the desired codon. For example, the reverse complement of NNK is MNN where M = C/A. For HNF1A, FOXG1 and FOXP1, which have DBD sizes <300 bp (100 amino acids), mutants were incorporated by PCR using oligonucleotide pools either as templates, 5′ primers (20–60 bp) or 3′ primers (20–60 bp). All 5′ ends of the forward and reverse primers, as well as the corresponding oligonucleotide pools for these three genes, shared constant regions to facilitate restriction enzyme digestion for downstream cloning (Supplementary Table 1). Each PCR reaction was performed using NEB Q5 Hot-start polymerase for ten cycles, followed by ExoSAP treatment to remove single-stranded oligonucleotides and purification using the MinElute kit.

All three TF DBD WT sequences were PCR-amplified from the MORF library (Addgene, cat. no. 192821). Motif sequences were ordered as oligonucleotides from Sigma-Aldrich, flanked by constant regions containing *Eco*RI and *Not*I sites for cloning into the pH3U3-zif268 (omega) backbone (Addgene, cat. no. 18046).

### Plasmid library preparation

To clone TF DBDs into the B1H assay vector, we used the vector backbone of pB1H2w2-zif268 (Addgene, cat. no. 18045), between the two restriction enzyme sites *Kpn*I and *Xba*I by digestion and ligation with T4 DNA ligase following the manufacturer’s protocol. The binding motif was cloned into pH3U3-zif268 (omega) (Addgene, cat. no. 18046), between the two restriction enzyme sites *Not*I and *Eco*R1, using HiFi ligation following the manufacturer’s protocol for single-strand-double-strand ligation. For the abundance assay, TF DBDs were cloned into the plasmid backbone PTB182, which was acquired from the Bardwell laboratory^55^ and modified for flexible cloning in the Lehner laboratory.

In detail, each separate PCR reaction was performed for ten cycles, followed by purification and cloning into plasmid backbones using *Xba*I and *Kpn*I restriction sites. The PCR products were inserted into the *Xba*I–*Kpn*I-linearized vector pB1H2w2-zif268 (ref. ^53^) digested with the same restriction enzymes. The PTB182 vector was linearized using PCR by introducing *Xba*I and *Kpn*I sites between the two Gly-Ser linkers, followed by enzymatic digestion and purification to prepare it for ligation.

T4 ligation was followed by transformation into NEB5α competent cells according to the manufacturer’s instructions. After recovery in 1 ml SOC medium, a 1-µl aliquot was diluted into 50-µl LB and plated on LB agar plates containing ampicillin, and the remainder was inoculated into 10 ml LB medium containing ampicillin for overnight culture. Colony counts from the plates were used to estimate the number of independent transformants. Libraries with at least 20-fold higher transformant numbers than variant counts were considered to have sufficient coverage. Plasmids were then prepared from the liquid culture, and the three same-gene mutant plasmid libraries were combined in equimolar amounts.

### DNA-binding selections

The B1H assay was adapted from Meng and Wolfe^52^, with key modification of using liquid rather than plate-based selection. *E.* *coli* DH5 alpha USO hisB^−^ pyrF^−^ rpoZ^−^ cells (Addgene, cat. no. 18044) were made electrocompetent in-house and first transformed with motif-carrying plasmids. A colony with a validated plasmid was expanded, made electrocompetent again and transformed with 100 ng of the library plasmid. An aliquot of recovery medium was plated on 2 × YT containing carbenicillin and kanamycin to estimate transformant numbers; the remainder was cultured in 25 ml 2 × YT containing carbenicillin and kanamycin for each biological replicate.

After overnight growth (achieving >100× transformant coverage), 5 ml of culture was washed five times in water, incubated for 1 h at 37°C in NM medium (no 3-amino-1,2,4-triazole), and finally inoculated into NM medium with carbenicillin, kanamycin, isopropyl β-d-1-thiogalactopyranoside as in the original protocol and 1 mM 3-amino-1,2,4-triazole. Cultures were shaken at 37°C for 24 h, with aliquots measured in 96-well plates (three technical replicates) for real-time optical density monitoring, including WT and negative controls. The remaining wash medium was spun to collect input cell pellets for storage at −20°C. After 24 h, output cell pellets were collected, and plasmid DNA was extracted along with input samples.

### Abundance selections

For the STS assay^55^, plasmids were electroporated into *E.* *coli* DH5α (NEB 5α) electrocompetent cells. As with B1H, aliquots were plated to estimate transformant numbers; the rest was cultured in 25 ml LB containing ampicillin. After confirming >100× variant coverage, 50 μl of overnight culture was seeded into LB containing ampicillin with serial spectinomycin concentrations (diluted from 1,000 mg μl^−1^ stock at 0.75× intervals). Three biological replicates were assigned to three consecutive concentrations across 21 dilutions. After 5-h shaking at 37°C, OD was measured and pellets collected for plasmid preparation.

### Sequencing library preparation

Library preparation used primers specific to flanking regions of the mutated region, with 5′ overhangs for Illumina indexing. First PCR (eight cycles) was followed by 1.0× SPRI bead clean-up, then indexed primer PCR (ten cycles) and 0.8× SPRI selection. Libraries were pooled equimolarly and sequenced on Illumina NovaSeq 6000 S2 flow cell (PE250) at the Wellcome Sanger DNA pipeline.

### Sequencing data processing

Raw read counts were processed with DiMSum v1.3.2^90^ using the following parameter settings: mutagenesisType = codon, fitnessMinInputCountAll = 10, vsearchMinQual = 20 and fitnessDropoutPseudocount = 0. For abundance assays, the half-maximal inhibitory concentration was estimated using the drc R package. However, half-maximal inhibitory concentration fitting was limited by poor signal in dead mutants, reducing usable data. Instead, we selected three consecutive concentrations showing optimal separation between synonymous and nonsense variants, balancing resolution and noise. Scores were averaged across these biological replicates without weighting (as the shared inputs would penalize low-fitness variants due to Poisson error).

Abundance assay scores were normalized against the mode of synonymous and nonsense variants for each selected condition and then averaged. Binding assay scores were similarly normalized using the mode of synonymous and nonsense, then averaged across biological replicates.

### Structural analysis

All three TF DBDs bound to the corresponding motifs in the assay were modeled using AlphaFold 3^63^, and compared to the closest solved structures in the PDB^60,64,72,74,91^ database. AlphaFold 3 predictions were used as a reference for visualization and analysis, including distance to DNA,rSASA calculation and pocket identification, to be consistent across all the TFs in our assay, since not all TFs have solved structures bound to DNA, nor all the mutated residues present in the solved structures. For instance, the structures of HNF1A bound to DNA are with different DNA sequences and have some residues not solved or not included in the solved structures^60,64^. There is no DNA-bound structure for FOXP1, and we used a highly conserved FOXP2-bound DNA (PDB 2a07)^74^ as a proxy.

Core residues were defined as those with rSASA <25%, DNA-contacting residues as those with ΔrSASA >1% (bound versus unbound), and all others as surface residues. rSASA was computed using the built-in function in Pymol.

Residues defined as DNA-contacting by ∆rSASA (∆rSASA-based DNA contact) included any putative DNA interface contacts to avoid bias in modeling the contribution of protein abundance to DNA binding.

Curated TF–DNA contacts were analyzed and visualized using DNAproDB^59^. Euclidean distances to DNA were also calculated from the predicted DNA–protein complex using the ChimeraX ‘contacts’ function with default parameters and a Van der Waals overlap threshold of <0.4 Å. All 3D structure visualization was performed with ChimeraX-1.5.

Structural alignment of FOXG1 and FOXP1 DBD monomers was conducted using the ChimeraX ‘Matchmaker’ function, using the ‘Chain pairing’ and ‘Best-aligning pair of chains’ options. Sequence alignment was performed with the BLASTP ‘Blast 2 Sequences’ tool^92^.

Secondary structure annotation was performed using SSDraw^93^ on the AlphaFold 3-predicted structure, with coloring based on the pLDDT (the predicted local distance difference test) confidence scores, where 100 is the highest confidence and 0 is the lowest confidence in local structure prediction.

### Binding residuals

To assess deviations from the expected relationship between abundance and binding, a LOESS regression model was fitted to predict binding scores as a function of abundance using mutations at positions that do not contact DNA.

We used the loess() function in R^94^, set the Span parameter to 0.9 and used weights as the inverse variance of binding scores. Two focal points (0,0) and (−1, −1) were added with weights = 1 × 10^4^, so that LOESS curves pass through the two focal points.

We excluded DNA-contacting residues when fitting the LOESS regression because the regression is intended to model the a priori expectation that a mutation reduces DNA‑binding activity solely through its effects on protein abundance. Nevertheless, for comparison, we also fitted a LOESS regression to all data (including DNA‑contacting residues) while keeping all other parameter settings and methods consistent (Extended Data Figs. 1h and 7b).

Residuals were calculated by subtracting the binding scores predicted by the LOESS model, based on the abundance scores, from the observed binding scores.

Per-position median absolute residuals were calculated by taking the median of all absolute residuals at each position, after excluding variants with very low abundance scores, which typically correspond to premature stop codons phenotypes (that is, abundance less than −0.8). We excluded variants with very low abundance scores (abundance less than −0.8), rather than only stop codons, to objectively identify positions amenable to perturbation (‘engineerable’) for later overlay with pockets. If all missense variants at a position fall below this threshold, the position is considered essential and unsuitable for perturbation; if at least one missense variant retains substantial abundance, we calculate a quantitative median residual from those variants that are not expected to behave like stop codons.

### Allosteric decay

We used the nlsLM function from the minpack.lm package in R to fit an exponential decay model to the absolute binding residuals as a function of the minimum distance between each protein residue and DNA. The model followed the form: *y* = *a* × exp(*−b* × *x*) *+* *c*, where *y* represents the absolute residual and *x* the distance. Initial parameter values were set as follows: *a* was initialized to the maximum absolute residual, *c* to the minimum and *b* to 0.5.

### Pocket prediction

Among the available tools, we selected PASSer (Protein Allosteric Sites Server)^95,96^ due to its comprehensive integration of several algorithms, detailed reporting on the pockets and capability for easy visualization. Pocket identification and druggability prediction within PASSer is based on FPocket^66^, druggability scores ranging from 0 to 1. 0 indicate unlikely to be bound by small molecules or peptides, and a score of 0.5 or above is generally considered a good, potentially druggable pocket.

We used the web server ensemble model, defining one Chain for pocket identification by uploading the AlphaFold 3-predicted structure with all the mutated regions and the DNA included. Resulting pocket PDB files were visualized in ChimeraX.

### Protein stability prediction

To predict the effects of amino acid substitutions on protein folding stability, ThermoMPNN^97^, a deep learning model that estimates changes in folding free energy (ΔΔG), was used. All predictions utilized the default parameters of the ThermoMPNN Colab notebook (https://colab.research.google.com/drive/1OcT4eYwzxUFNlHNPk9_5uvxGNMVg3CFA). The AlphaFold 3-predicted model of the HNF1A DBD (AF-P20823-F1) was used. For FOXG1 and FOXP1, the crystal structures PDB 7CBY and PDB 2KIU were used, respectively. Input structures were prepared by removing water molecules and heteroatoms. ThermoMPNN was then applied to scan all possible single amino acid substitutions across each domain, including cysteine residues.

### Clinical variant interpretation

Variant function classification was based on the following criteria: mutations were classified as neutral if the effect size was ≤0.1 and BH FDR ≥ 0.05; as loss-of-function if the binding score was <−0.1 and FDR < 0.05 and as gain-of-function if the binding score was >0.1 and FDR < 0.05. Variants with large effect sizes that were not statistically significant, or with small effect sizes that were statistically significant, were considered uncertain (Extended Data Fig. 5e).

Biophysical classification considered mutations as abundance-affecting if the residuals were ≤0.1 (unless classified as neutral) and included ‘dead’ variants with abundance scores below −0.8. Mutations were considered binding-affecting at the DNA interface if residuals were >0.1, FDR < 0.05 and located at DNA-contacting positions (Extended Data Fig. 5f). Allosteric binding-affecting mutations were defined as those meeting the same criteria but located outside DNA-contacting positions (Extended Data Fig. 5f).

### Statistical tests

All statistical analyses were performed using R. To assess whether the functional scores (binding and abundance, respectively) of each variant significantly differ from zero across biological replicates, we performed one-sample *t*-tests. Resulting *P* values were corrected for multiple testing using the BH FDR method within each dataset.

To evaluate whether individual residue positions or specific groups of residues (for example, core, surface, etc.) differ significantly from zero, we used one-sample Wilcoxon signed-rank tests. For datasets with fewer than 30 samples, Bonferroni correction was applied; for those with 30 or more samples, BH correction was used. To compare variance between groups, we performed Kruskal–Wallis tests, followed by post hoc Dunn’s tests for pairwise comparisons.

### Reporting summary

Further information on research design is available in the Nature Portfolio Reporting Summary linked to this article.

## Online content

Any methods, additional references, Nature Portfolio reporting summaries, source data, extended data, supplementary information, acknowledgements, peer review information; details of author contributions and competing interests; and statements of data and code availability are available at https://doi.org/10.1038/s41594-026-01908-2.

## Supplementary information

Reporting Summary

Peer Review File

Supplementary Table 1 Oligonucleotide sequences.

## Source data

Source Data Fig. 1 Tabular source data.

Source Data Fig. 2 Tabular source data.

Source Data Fig. 3 Tabular source data.

Source Data Fig. 4 Tabular source data.

Source Data Fig. 5 Tabular source data.

Source Data Fig. 6 Tabular source data.

Source Data Fig. 7 Tabular source data.

Source Data Fig. 8 Tabular source data.

Source Data Extended Data Fig. 1 Tabular source data.

Source Data Extended Data Fig. 6 Tabular source data.

Source Data Extended Data Fig. 7 Tabular source data.

Source Data Extended Data Fig. 9 Tabular source data.

## Data Availability

All DNA sequencing data have been deposited in the European Nucleotide Archive (ENA) at EMBL-EBI under accession number PRJEB97482. The complete processed dataset is available via Zenodo at https://doi.org/10.5281/zenodo.17457955 (ref. ^98^).Data and materials can be obtained from the corresponding authors upon request. Source data are provided with this paper.

## Extended data

**Extended Data Table 1:** Pockets

PocketTF_Pocket	Type	Sum of Absolute Binding-residuals	Druggability score	AA positions
HNF1A_1	**Distant**	1.79	0.247	A217, E228, E235, F268, L232, P224, Q221, R220, T231
HNF1A_2	**Distant**	0.62	0.108	G253, L254, Q250, Q252, R244, S249, V246
HNF1A_3	Interface	0.43	0.003	E261, N266, R229, V262, Y265
HNF1A_4	Interface	0.56	0.001	E274, F204, F215, Q211, R263, R271, W267
HNF1A_5	**Distant**	0.19	0.001	A251, E234, N237, P248, R238
HNF1A_6	Interface	1.01	0.000	K205, R203, W206
FOXG1_1	Interface	0.44	0.420	E220, N221, Y186, Y217, Y218
FOXG1_2	Interface	1.79	0.070	G252, K251, L204, N205, N253, R244, T203, Y208
FOXG1_3	Interface	0.83	0.000	F239, K241, L233, S234, W255
FOXP1_1	Interface	1.83	0.494	A500, N505, R504, T508, W509, Y470, Y501
FOXP1_2	Interface	0.57	0.124	A532, G531, K530, N528, Q485, T487, V533
FOXP1_3	Interface	1.81	0.048	L488, L517, R514, R525, S518, W534
FOXP1_4	Interface	0.45	0.041	A477, N493, R497, W494
FOXP1_5	**Distant**	1.39	0.013	E480, F468, F541, L479, Q476, R475, R545, S472
FOXP1_6	Interface	3.04	0.007	A471, C522, H520, N516, R544, T469
FOXP1_7	**Distant**	1.14	0.002	G551, I549, K548, P546, Q542, Q547
FOXP1_8	**Distant**	0.57	0.001	E483, E490, L486, P482, S481

## Code availability

Code is available via GitHub at https://github.com/lehner-lab/TF-MAPS.
