# Supplementary material for: TF-MAPS: fast high-resolution functional and allosteric mapping of DNA-binding proteins
Source: Nat Struct Mol Biol. 2026 Nov 11;33(11):1704–17. doi: 10.1038/s41594-026-01908-2 (PMC13759329; doi:10.1038/s41594-026-01908-2)
Supplement: Supplementary file 2 [file 41594_2026_1908_MOESM2_ESM.pdf]

nature portfolio

Peer Review File

TF-MAPS: fast high-resolution functional and allosteric mapping of DNA-binding proteins

Corresponding Author: Professor Ben Lehner

This manuscript has been previously reviewed at another journal. This document only contains information relating to versions considered at Nature Structural & Molecular Biology.

Version 0:

Decision Letter:

18th Mar 2026

Dear Dr. Lehner,

Thank you again for submitting your manuscript "TF-MAPS: fast high-resolution functional and allosteric mapping of DNA-binding proteins" to the journal. I sincerely apologize for the unusual delay in responding, due to a third reviewer having to be recruited at a late stage in the peer review process. We now have comments (below) from the 3 reviewers who evaluated your paper. In light of those reports, we remain interested in your study and would like to see your response to the comments of the referees in the form of a revised manuscript.

You will see that while the reviewers appreciate the work, reviewer #1 requests better justification and description of the LOESS regression approach, determination of whether known pathogenic variants map to or near these pockets and clarification of the therapeutic relevance of the identified pockets. Moreover, reviewer #2 requests showing a correlation between the abundance scores with another type of low-throughput measurement of a selected few variants' abundance across the range of abundance scores (such as by Western blot or tagging with fluorescence protein and flow cytometry), discussion of the possibility that mutations could affect PPIs or PTM sites rather than exclusively DNA contact sites or allosteric effects, and discussion of the possibility of C-end degron effects. Lastly, reviewer #3 requests discussion of limitations of the study to single site analysis as opposed to combinatorial space, discussion of other types of TF interactions with allosteric communication, and elaborating on the comparison between TF-MAPS and other methodologies, as well as streamlining some of the data presentation in the figures for clarity.

Please be sure to address/respond to all concerns of the referees in full in a point-by-point response and highlight all changes in the revised manuscript text file. We expect to see your revised manuscript within 2 months. We are committed to providing a fair and constructive peer-review process; please do not hesitate to contact us if you would like to discuss the reviews or anticipate any delay with the resubmission. If you cannot send it within this time, please contact us to discuss an extension; we will still consider your revision provided that no similar work has been accepted for publication at NSMB or published elsewhere.

REPORTING AND DATA AVAILABILITY: Please provide updated files as follows with your revision. As you already know, we put great emphasis on ensuring that the methods and statistics reported in our papers are correct and accurate.

1. REPORTING SUMMARY: Please provide an updated file:

https://www.nature.com/documents/nr-reporting-summary.pdf

This form is a dynamic 'smart pdf' and must be downloaded and completed in Adobe Reader.

2. REPORTING OF STRUCTURAL DATA: If there are additional or modified structures in the revision, please submit the

corresponding PDB validation reports with your other files, and use the figshare integration system to provide access to maps, half-maps, and models.

Manuscripts reporting new structures should contain a table summarizing structural and refinement statistics. Please include these tables for new cryo-EM or -ET (https://www.nature.com/documents/nr-tables-cryo-em.doc and modifying this file for ET), NMR (https://www.nature.com/documents/nr-tables-nmr.doc) and X-ray crystallography (https://www.nature.com/documents/nr-tables-xray.doc). To facilitate assessment of the quality of the structural data, a stereo image of a portion of the electron density map (for crystallography papers) or of the superimposed lowest energy structures (>10; for NMR papers) should be provided with the submitted manuscript. If the reported structure represents a novel overall fold, a stereo image of the entire structure (as a backbone trace) should also be provided. For cryo-EM structures, a representative micrograph showing individual particles should be provided in the figures alongside a processing workflow (please see our Editorial for more information: https://www.nature.com/articles/s41594-025-01567-9).

DATA DEPOSITION: We require deposition of coordinates (and, in the case of crystal structures, structure factors) into the Protein Data Bank with the designation of immediate release upon publication (HPUB). Electron microscopy-derived density maps and coordinate data must be deposited in EMDB and released upon publication. Deposition and immediate release of NMR chemical shift assignments are requested. To avoid delays in publication, dataset accession numbers must be supplied with the revised manuscript and appropriate release dates must be indicated at the galley proof stage.

3. REPORTING OF LIGHT MICROSCOPY DATA: For any revision that includes light microscopy data, we ask our authors to please include a completed light microscopy reporting table [https://www.nature.com/documents/Light_microscopy_reporting_table.xlsx] to ensure the methods are described thoroughly. The table will be available to reviewers and

ultimately published, should the manuscript be accepted at the journal. When submitting the revised version of your manuscript, please pay close attention to our href="https://www.nature.com/nature-portfolio/editorial-policies/image-integrity">Digital Image Integrity Guidelines.

4. CODE AND COMPUTATIONAL WORK: If newly developed, unpublished code was used in this work, it must be provided with the revision and access to it must be disclosed in the Code Availability Statement in the manuscript. Please also provide the completed software submission checklist. This form is a dynamic 'smart pdf' and must be downloaded and completed in Adobe Reader, instead of opening it in a web browser: https://www.nature.com/documents/nr-software-policy.pdf

If molecular dynamics (MD) simulations were performed, please refer to this Editorial and provide a completed MD simulations checklist with your revision: https://www.nature.com/articles/s42003-023-04653-0

Lastly, if it applies, please complete and upload the completed machine learning checklist with your submission for re-review: https://www.nature.com/documents/machine-learning-checklist.pdf

5. SOURCE GEL IMAGES: Unprocessed scans should be provided for all gels and western blots presented in figures and should be clearly labelled with the figure number in the file title. The source gel images should be uncropped, unmodified images, with molecular weight markers. Uncropped, unmodified gel images from existing experimental repeats, in addition to the data shown in the figures, can also be presented in source data. Please provide these files as PDF with your revision.

Please ensure that all control panels for gels and western blots are appropriately described as loading or IP controls. Lastly, please ensure that all images in the paper are checked for duplication of panels and for splicing of gel lanes.

6. SOURCE NUMERICAL DATA: we urge authors to provide, in tabular form in excel files, the data underlying the graphical representations used in figures. This is to further increase transparency in data reporting, as detailed in this editorial (http://www.nature.com/nsmb/journal/v22/n10/full/nsmb.3110.html). Please provide one excel file per figure max, with one panel per tab. When submitting files, the title field should indicate which figure the source data pertains to.

7. DATA AVAILABILITY: this journal strongly supports public availability of data. All data used in accepted papers should be available via a public data repository, or alternatively, as Supplementary Information. If data can only be shared on request, please explain why in your Data Availability Statement and in the correspondence with your editor.

For some data types, deposition in a public repository is mandatory as detailed below:

https://www.nature.com/nature-research/editorial-policies/reporting-standards#availability-of-data

Finally, please ensure that you retain unprocessed data and metadata files after publication, ideally archiving data in perpetuity, as these may be requested during the peer review and production process or after publication if any issues arise.

EXTENDED DATA FIGURES

When re-submitting your manuscript, please ensure that any supplementary figures and tables that are crucial to the manuscript's conclusions are converted into Extended Data figures and tables to increase visibility of these data. Extended Data figures and tables are online-only (present in the online PDF and full-text HTML versions of the paper), peer-reviewed display items that provide essential background to the article but are not included in the main article due to space constraints. A maximum of ten Extended Data display items (figures and tables) is permitted.

While we encourage the use of color in preparing figures, please note that this will incur a charge to partially defray the cost of printing. Information about color charges can be found at http://www.nature.com/nsmb/authors/submit/index.html#costs

Nature Structural & Molecular Biology is committed to improving transparency in authorship. As part of our efforts in this direction, we are now requesting that all authors identified as 'corresponding author' on published papers create and link their Open Researcher and Contributor Identifier (ORCID) with their account on the Manuscript Tracking System (MTS), prior to acceptance. This applies to primary research papers only. ORCID helps the scientific community achieve unambiguous attribution of all scholarly contributions. You can create and link your ORCID from the home page of the MTS by clicking on 'Modify my Springer Nature account'. For more information please visit please visit www.springernature.com/orcid.

Please use the link below to submit your revised manuscript and related files:

Link Redacted

Note: This URL links to your confidential home page and associated information about manuscripts you may have submitted, or that you are reviewing for us. If you wish to forward this email to co-authors, please delete the link to your homepage.

We look forward to seeing the revised manuscript and thank you for the opportunity to review your work.

Sincerely,
Sara

Sara Osman, Ph.D.
Senior Editor
Nature Structural & Molecular Biology

Reviewers' Comments:

Reviewer #1 (Remarks to the Author):

The authors present TF-MAPS (Transcription Factor-Mutational Allosteric Propagation Scan) as a high-throughput approach for comprehensively mapping how mutations affect DNA binding across transcription factor DNA-binding domains (DBDs), distinguishing direct interface effects from allosteric effects. The approach combines bacterial one-hybrid (B1H) assays to measure DNA-binding activity with spectinomycin tripartite sandwich (STS) assays to measure protein abundance, quantifying ~1,700 missense variants per protein across three disease-relevant TF DBDs (HNF1A, FOXG1, FOXP1). To infer allosteric effects, the authors use LOESS regression fitted to non-DNA-contacting residues and calculate binding-residuals to quantify mutational effects on DNA binding beyond those mediated by changes in protein folding/stability. Using this approach, they generate the first comprehensive allosteric maps for TF DBDs, revealing expanded DNA binding interfaces, distance-dependent decay of allosteric effects, anisotropic allosteric hotspots, and potentially druggable surface pockets.

Critique

The main points of the manuscript as stated in the subtitles are generally supported by the provided data. However, additional information and clarification would strengthen the conclusions and improve the paper's rigor.

1. The LOESS regression approach is foundational to this study, yet its justification and limitations are inadequately described. The paper states that LOESS is fitted "using mutations at positions that do not contact DNA" (Methods, lines 645-647), but does not explain why this exclusion is essential or what consequences arise from including these residues. The authors should demonstrate how binding-residuals are affected by the inclusion or exclusion of these mutations in the LOESS regression, and clarify the extent to which structural data is required for accurate calculation of binding-residuals.

2. The paper states that "FOXG1 and FOXP1 have the largest number of known pathogenic variants among the 43 human

forkhead family TFs" (lines 113-114). However, despite the paper's claim that TF-MAPS provides "reference atlases for clinical genetics" (lines 484-485), no mechanistic classification of FOXG1 or FOXP1 pathogenic variants is presented, in contrast to the comprehensive analysis provided for HNF1A (Fig 6). Given that the paper identifies druggable pockets in both proteins (Fig 9) and suggests they should be targeted therapeutically (lines 465-467), it is important to determine whether known pathogenic variants map to or near these pockets. If pathogenic variants are absent from these pockets, the authors should clarify the therapeutic rationale: does pocket druggability alone justify therapeutic development, or is the proposed mechanism allosteric modulation of distant disease-causing variants? This analysis would clarify both the therapeutic relevance of the identified pockets and the proposed mechanism of action for pocket-targeted drugs. Additionally, comparing pathogenic variant distributions between these homologous proteins would provide insights into whether disease mechanisms are conserved or divergent across TF families.

Minor Issues:
Figure 5c legend: Typo - "corresponding ot the structural annotation" → "corresponding to the structural annotation"
Extended Data Figure 2d legend: First two sentences are redundant
Figures 8 and 9 are not labelled

Reviewer #2 (Remarks to the Author):

In this work, Li and Lehner introduce TF-MAPS (Transcription Factor-Mutational Allosteric Propagation Scan), a high-throughput method to map residues that affect DNA binding through abundance changes, contact with DNA or allosteric effects. They implement the method to generate maps for the DNA-binding domains (DBDs) of Hepatocyte Nuclear Factor 1 homeobox Alpha (HNF1A) and two forkhead box (FOX) transcription factors (TFs), FOXG1 and FOXP1.

To evaluate the DNA binding capacity of the three DBDs, Li and Lehner employed bacterial one hybrid (B1H) using HIS3 as the reporter gene. In parallel they evaluated their cellular abundance using the spectinomycin tripartite sandwich (STS) assay. By utilizing deep mutational scanning approach, most single amino acid substitution variants and nonsense variants were screened. To disentangle changes in DNA binding due to abundance from changes due to a role in DNA binding they used LOESS (Locally Estimated Scatterplot Smoothing) regression to estimate the expected changes in binding due to abundance and then calculated the residuals between the expected and observed binding of each variant to deduce their contribution in binding independent of their abundance.

They find that the binding residuals are largest for the residues in contact with DNA and decay exponentially with increasing distance from the DNA but also vary significantly based on the kind of amino acid substitution and the exact residue in the WT sequence. Based on the residuals between the expected (based on the exponential decay model) binding residual and the observed ones, they were able to map “allosteric hotspots”. They use this knowledge along with the FPocket algorithm to identify potential targets for small molecule drugs.

The approach of combining a protein abundance screen with some sort of functional screen to draw conclusions regarding the mechanism behind the phenotype of protein variants (eg Matreyek et al., Genome Medicine, 2021 - PMID: 34649609) is not novel. Similar attempts on characterizing the effect of mutations on TFs using yeast one-hybrid have also been performed before (McDonnell et al., Molecular Systems Biology, 2024 - PMID: 38849565). However, to my knowledge the combination of an abundance screen with a DNA binding screen and the accompanying computational analysis to infer which TF residues contribute to allosteric effects is indeed novel. The method presented in this work will help with the interpretation of variant effects and with the identification of druggable pockets for the design of new small molecule drugs.

Overall, this is a rigorously executed study, and the data are robust. The results and methodology are presented clearly, and careful implementation of proper statistics contribute to a high confidence in the presented results.

- It is impressive and interesting to examine the data of the abundance screen with 21 different concentrations of spectinomycin. To add to it, I believe it would significantly increase the confidence in the abundance scores if a correlation was shown between the abundance scores and some sort of low-throughput measurement of a selected few variants’ abundance covering the entire range of abundance scores (eg Western blot or tagging with fluorescence protein + flow cytometry). Given the non-linear correlation of the replicates in different conditions and the Extended Data Fig. 7k, showing a non-linear correlation between the Tm change of five variants and their abundance scores, I believe it would be useful for the interpretation of the results to show how exactly the abundance scores correlate with such low-throughput measurements.
- A basic assumption used for the interpretation of the results is that mutations that change the DNA binding more than the expected (according to the change of abundance) must affect either DNA contact sites or act through some allosteric effect. How do we know that such a mutation does not increase the interaction with some endogenous protein or introduce a site of post-translational modification thus potentially inhibiting DNA binding indirectly? Perhaps this possibility should be addressed in the manuscript.
- According to the Addgene vector 18045 map, the TF DBDs appear to have been introduced at the C-terminus of the RNA polymerase subunit for the B1H screen. If that is right, could the fact that the C-terminus was exposed contribute to C-end degron effects that might not be present in the context of the STS assay, where the C-terminus is not exposed? If that is the case the interpretation of the variants near the C-terminus could drastically change when using the method in bacteria (Cragan et al., Journal of Biological Chemistry, 2025 - PMID: 40023398), yeast (Kong et al., Nature Communications, 2023 -

PMID: 38102142) or human cell lines (Koren et al., Cell, 2018 - PMID: 29779948, Voutsinos et al., biorxiv 2025 - PMID: 40463067). Could the authors discuss this possibility as a limitation of the method in this work?

• Sometimes “TF” is used when referring to DBDs, especially in figures. I think for clarity it would be better if “DBD” or “TF-DBD” is used instead, given that only these domains of the TFs are screened.

• Why were the variants of very low abundance scores excluded in the calculation of the per position median absolute residuals and not simply the stop codon ones? (lines 653-655).

• The bacterial species used in the study should be mentioned in the methods.

Minor comments:

• Line 45-46: “DNA” should go before “nuclear localisation” for the verb “binding” to refer to it.
• Line 547: Why four DBDs, aren’t there only three?
• Line 549: “backbone” font is different
• Line 681: Is “80” a typo?
• Are references 28 and 52 the same?
• In Figure 1h I would change the colour of the blue rho (all) as it might be confused for the rho of the blue dots (DNA contact).
• In the heatmaps of Figure 2 and Figure 7 the amino acids on the vertical axes are not well aligned to the map.
• In Figure 6c there is a red line on the black block of the likely pathogenic and in 6d the number 43 is slightly out of the grey block.
• In Extended Data Figure 4a, what is the red dotted line?

Reviewer #3 (Remarks to the Author):

The work of Li and Lehner aim to characterize the mutational landscape in transcription factors that leads to effects in binding specificity, independently of protein concentration in a high throughput manner. The methodology called Transcription Factor-Mutational Allosteric Propagation Scan (TF-MAPS), uses deep mutational scanning (DMS) and an STS assay to identify the effects of mutations on folding and not binding. After proper normalization and modeling of the expected effect of protein concentration the authors are able to compute binding-residual values that are proxy of how detrimental or enhancing is a mutation in a given residue position of a TF. The authors focus on DNA binding domains and apply their methodology in a bacterial system. Li and Lehner apply TF-MAPS to 3 different proteins and are able to verify the effect of mutations at the binding interface, estimate the decaying distant-dependent effect of mutations near the binding interface, as well as characterize those allosteric hotspots that have larger effect that expected. The authors also investigate and contrast with clinical variants and provide some mechanistic interpretation of these mutations using the binding-residual scores and its statistics as interpretative tools. Finally, the authors also identify relevant drugabble hotspots and propose some plausible sites and regions where druggability and regulation of TF binding might be possible. The authors support the idea that TF-MAPS is general and scalable as well as fast.

In my opinion, this is an interesting study that is well motivated and carefully designed to isolate the direct effects of mutation on DNA recognition by TFs. Although the use of DMS to study mutational landscapes is not new, the combination with the folded protein abundance STS assay is innovative and leads to interesting results. In particular, I appreciated the effort to validate as many mutations as possible that do have stronger residuals either via structural interpretations, literature review and modeling. This careful validation brings validity to other non-expected interpretations of the effects of mutations. I also appreciate the idea of comparing homologous TFs like FOXG1 and FOXP1, the results showcase how similar TFs that are members of the same family and conserve their interaction interfaces can have very distinct responses to mutations, showcasing the evolutionary diversity and complexity of these systems. Therefore my assessment is that both the specific results and the general methodology of TF-MAPS are valuable and has substantial contributions to the field. I see proper use of statistics and treatment of uncertainties as well as appropriate citations of prior work. My main points focus on the presentation of results and the need to do some clarifications on the limitations and effectiveness of this technique. I will enumerate those comments first in a general sense and then give some specific points to improve the clarity of the manuscript.

General Comments

1. One important aspect that needs to be clearly stated is that this methodology only considers single site mutations. Even though DMS leads to a large amount of variants, there is still a much larger combinatorial space where epistatic interactions might dominate and alter both direct binding and allosteric effects. Authors should comment on this and clarify this limitation, as the current manuscript leads to the idea that the landscapes obtained are global and “comprehensive”.

2. The title of the manuscript as well as the name of the methodology highlights the concept of allostery, yet a big part of the results focus on direct effect on the TF-DNA interface. Although important for validation, I left thinking that more analysis or focus should be done to the allosteric hotspots. For example, I am curious if there are any allosteric hotspots that have a larger residual effect than interfacial mutations and if so, a potential interoperation might be valuable.

3. Many transcription factors have allosteric communication between a sensing domain and the DBD that activates/deactivates them. Why did the authors not include this in their work? The authors do mention that in the future it would be valuable to include more TF domains, however, I feel that the more interesting cases of allostery are found when a sensing domain binds to a ligand that later induce DNA recognition. The authors should justify why this was not part of their

investigation and provide maybe a putative analysis of its importance.

4. Although it is evident that the authors did a comprehensive study of mutations, I feel their figures are too crowded. Many times the information needed to convey their message as explained in the text requires only a fraction of the data shown in figures. I suggest the authors to move redundant or non-discussed data on figures to the SI.

5. The authors highlight the “fast” nature of TF-MAPS. One argument provided is the fact that this is done in a bacterial system. However, it is not clear to me why this high throughput methodology is faster than other types of DMS based systems. Given that this is important for adoption, I suggest that the authors spend more time explaining or comparing why TF-MAPS is faster or more efficient than other methodologies.

Specific Comments

1. Page 6, line 191. The authors mention that the HNF1A binding of each variant against its abundance shows a strong non-linear correlation. I believe the wording is a bit confusing as it makes it sound like the correlation is strongly non-linear. I believe the authors refer to a strong correlation. Looking at the data, I don’t see a clear non-linearity and I don’t think it affects conclusions so I would probably only refer to it as a correlation or dependence. If the authors think the non-linearity is important then there are ways to quantify the non-linearity, one way is contrasting Spearman with Pearson correlations and show a big deviation.

2. Fig.1a is clean and simple but a bit hard to follow, I suggest to improve it for clarity.

3. I am not sure if the term “strict contacts” is the most appropriate for contacts based on Alphafold3, since those complexes are coming from a predictive algorithm. I would think that those coming from experimental structures should be called “strict” and those from AF3 perhaps “estimated”? Also in page 10, there is a reference to “direct contacts”, which makes this a bit more confusing. Perhaps I am missing something here, in any case it would be good to clarify.

4. For the predictive druggable pocket section, and more importantly for the ones with higher druggability scores, please provide a table or data file with the residues involving the pocket.

5. Page 13, line 480. Change “surface allosteric surfaces” with “allosteric surface”.

6. Page 16, Line 593. A single sentence paragraph is non-standard, I suggest merging with the previous paragraph.

Version 1:

Decision Letter:

Our ref: NSMB-A51934A

9th Jul 2026

Dear Dr. Lehner,

Thank you for submitting your revised manuscript "TF-MAPS: fast high-resolution functional and allosteric mapping of DNA-binding proteins" (NSMB-A51934A). It has now been seen by the original referees and their comments are below. The reviewers find that the paper has improved in revision, and therefore we'll be happy in principle to publish it in Nature Structural & Molecular Biology, pending minor revisions to satisfy the referees' final requests and to comply with our editorial and formatting guidelines.

We are now performing detailed checks on your paper and will send you a checklist detailing our editorial and formatting requirements in a couple of weeks. Please do not upload the final materials and make any revisions until you receive this additional information from us.

Thank you again for your interest in Nature Structural & Molecular Biology Please do not hesitate to contact me if you have any questions.

Sincerely,
Sara

Sara Osman, Ph.D.
Senior Editor
Nature Structural & Molecular Biology

Reviewer #1 (Remarks to the Author):

I appreciate the authors' efforts in addressing my comments. My questions and concerns have been adequately addressed, and I now support the publication of this manuscript.

Reviewer #2 (Remarks to the Author):

I have carefully reviewed the revised manuscript and the authors' response to the reviewers. My previous concerns/comments have been satisfactorily addressed. I believe the manuscript is appropriate for publication in its current form.

Reviewer #3 (Remarks to the Author):

I am satisfied with the responses in the rebuttal letter. My questions have been answered and the corresponding changes are acceptable. I also believe the responses to the other reviewers were appropriate and the modifications of the manuscript lead to a more clearly written study. I am supportive of this interesting and rigorous study.

Reviewer #3 (Remarks on code availability):

I did not run the code, but looked at the documentation and the code base. It seems appropriate although I cannot comment on reproducibility as I did not run it.

INTERNAL USE ONLY AIP modules required: ["Clinical","Life Science","Chemistry"]

Open Access This Peer Review File is licensed under a Creative Commons Attribution 4.0 International License, which permits use, sharing, adaptation, distribution and reproduction in any medium or format, as long as you give appropriate credit to the original author(s) and the source, provide a link to the Creative Commons license, and indicate if changes were made.
In cases where reviewers are anonymous, credit should be given to 'Anonymous Referee' and the source.
The images or other third party material in this Peer Review File are included in the article's Creative Commons license, unless indicated otherwise in a credit line to the material. If material is not included in the article's Creative Commons license and your intended use is not permitted by statutory regulation or exceeds the permitted use, you will need to obtain permission directly from the copyright holder.
To view a copy of this license, visit https://creativecommons.org/licenses/by/4.0/

Reviewers' Comments:

Reviewer #1 (Remarks to the Author):

The authors present TF-MAPS (Transcription Factor-Mutational Allosteric Propagation Scan) as a high-throughput approach for comprehensively mapping how mutations affect DNA binding across transcription factor DNA-binding domains (DBDs), distinguishing direct interface effects from allosteric effects. The approach combines bacterial one-hybrid (B1H) assays to measure DNA-binding activity with spectinomycin tripartite sandwich (STS) assays to measure protein abundance, quantifying ~1,700 missense variants per protein across three disease-relevant TF DBDs (HNF1A, FOXG1, FOXP1). To infer allosteric effects, the authors use LOESS regression fitted to non-DNA-contacting residues and calculate binding-residuals to quantify mutational effects on DNA binding beyond those mediated by changes in protein folding/stability. Using this approach, they generate the first comprehensive allosteric maps for TF DBDs, revealing expanded DNA binding interfaces, distance-dependent decay of allosteric effects, anisotropic allosteric hotspots, and potentially druggable surface pockets.

We thank the reviewer for their enthusiasm and very constructive comments.

Critique

The main points of the manuscript as stated in the subtitles are generally supported by the provided data. However, additional information and clarification would strengthen the conclusions and improve the paper's rigor.

1. The LOESS regression approach is foundational to this study, yet its justification and limitations are inadequately described. The paper states that LOESS is fitted "using mutations at positions that do not contact DNA" (Methods, lines 645-647), but does not explain why this exclusion is essential or what consequences arise from including these residues. The authors should demonstrate how binding-residuals are affected by the inclusion or exclusion of these mutations in the LOESS regression, and clarify the extent to which structural data is required for accurate calculation of binding-residuals.

In the revised Methods section (lines 759-763), we added the justification for excluding DNA-contacting residues from the LOESS regression and the potential consequences of their inclusion:

"We excluded DNA-contacting residues when fitting the LOESS regression because the regression is intended to model the a priori expectation that a mutation reduces

DNA-binding activity solely through its effects on protein abundance. Nevertheless, for comparison, we also fitted a LOESS regression to all data (including DNA-contacting residues) while keeping all other parameter settings and methods consistent (Extended Data Fig. 1h and 6l,m)."

Having said this, we repeated the analyses including the DNA contacting residues when fitting the loess curves and the results are extremely similar. These new analyses are shown in Extended Data Fig. 1g, h and Extended Data Fig. 6l, m. For the three proteins the binding residuals are extremely well correlated: r = 0.996, r = 0.997, and r = 0.969:

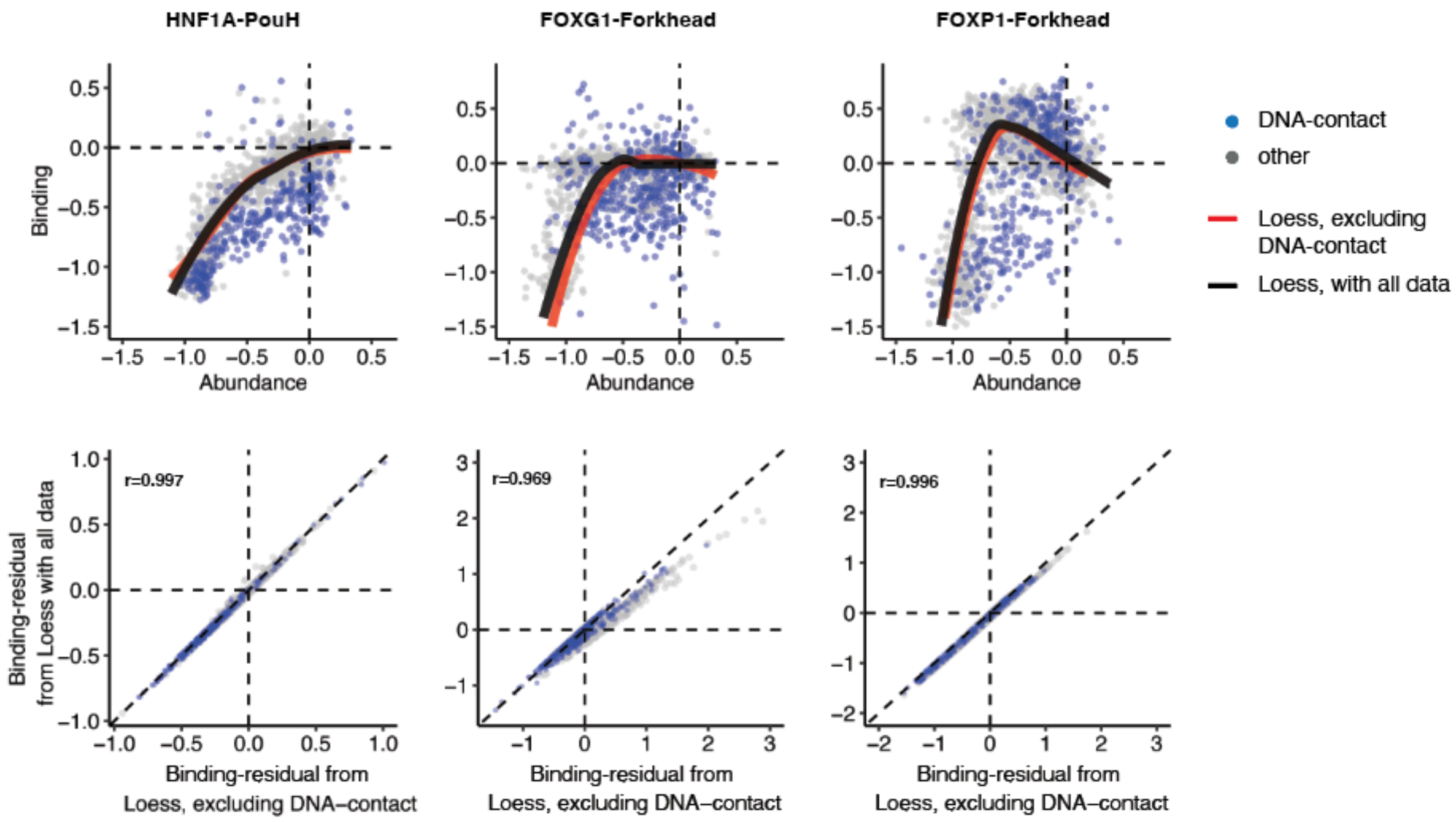

2. The paper states that "FOXG1 and FOXP1 have the largest number of known pathogenic variants among the 43 human forkhead family TFs" (lines 113-114). However, despite the paper's claim that TF-MAPS provides "reference atlases for clinical genetics" (lines 484-485), no mechanistic classification of FOXG1 or FOXP1 pathogenic variants is presented, in contrast to the comprehensive analysis provided for HNF1A (Fig 6). Given that the paper identifies druggable pockets in both proteins (Fig 9) and suggests they should be targeted therapeutically (lines 465-467), it is important to determine whether known pathogenic variants map to or near these pockets. If pathogenic variants are absent from these pockets, the authors should clarify the therapeutic rationale: does pocket druggability alone justify therapeutic development, or is the proposed mechanism allosteric modulation of distant disease-causing variants? This analysis would clarify both the therapeutic relevance of the identified pockets and the proposed mechanism of action for pocket-targeted drugs. Additionally, comparing pathogenic variant distributions between these

homologous proteins would provide insights into whether disease mechanisms are conserved or divergent across TF families.

We thank the reviewer for these suggestions. We have addressed the critique in three ways, which we believe have strengthened the manuscript:

(1) We have added a new Results section that presents a mechanistic classification of FOXG1 and FOXP1 variants. We have added Extended Data Fig. 9, which presents an analogous analysis of the mechanisms of pathogenic variants for FOXG1 and FOXP1. These results are presented at the end of the results section and also summarised in the discussion:

“In all three proteins approximately half (44-58%) the loss-of-function variants reduce protein abundance, between 19-39% are located in the binding interface, and the rest have an allosteric mechanism.”

And we have highlighted that additional mechanisms exist in the Discussion:

“Additional mechanisms of pathogenicity exist including changes in protein localization [64,83–85], post-translational modifications [86], and alternative splicing [87] that will not be captured by our platform.”

(2) We now show the positions of pathogenic/likely pathogenic residues on the 3D structures of FOXG1 and FOXP1, with those falling within the prioritised pockets (in all three cases, Pocket 1) highlighted (new Extended Data Fig. 5d for HNF1A, and Extended Data Fig. 9a,b for FOXG1 and FOXP1).

(3) Regarding pathogenic mutations within the target pocket and implications for drug design, the reviewer raises an excellent point. If pathogenic mutations occur in the pocket, they could affect small-molecule binding or the resulting modulatory effect. This is now mentioned in the Discussion.

Line 578:

“Finally, mutations in these pockets can reduce or increase DNA binding (Extended Data Figs. 5b, 8), indicating that small-molecule binding could be used to inhibit or enhance activity. For TFs with mutations within these pockets, mutation-specific small molecules may be necessary to modulate the effects. ”

The general therapeutic rationale is to modulate TF–DNA binding using small molecules that target these pockets. A high druggability (also known as ‘ligandability’) score does not justify therapeutic development, because binding to

many pockets will not modulate protein function (here, DNA binding). Identifying functional pockets is therefore crucial.

Importantly, both loss- and gain-of-function mutations are clinically relevant for all three TFs: HNF1A LoF causes MODY3 and hepatocellular adenoma, while HNF1A GoF protect from diabetes but increase hepatically derived lipoprotein risk factors for atherosclerosis and coronary artery disease (DeForest et al., Cell Genom 2023); FOXG1 and FOXP1 loss-of-function mutations cause severe neurodevelopmental disorders called FOXG1 syndrome and FOXP1 syndrome, while FOXP1 GoF is oncogenic (Walker et al., Science Signal 2015) and increased FOXG1 activity causes a distinct neural pathology (Wong et al., Intl J. Mol Sci 2019; Brunetti-Pierri et al., Eur J Hum Genet. 2011; Brunetti-Pierri et al., Eur J Hum Genet. 2012;). Thus, modulating DNA binding in either direction via these pockets could be therapeutically desirable, depending upon the indication. This has been better explained in the Introduction.

Lines 106 and 119:

"Gain of Function mutations in HNF1A have also been reported to protect from diabetes but increase hepatically derived lipoprotein risk factors for atherosclerosis and coronary artery disease [42]."

"Duplications of FOXG1 and FOXP1 are also pathogenic. FOXG1 duplications cause a distinct neural pathology from loss-of-function mutations [46,50], whereas FOXP1 duplications promote oncogenesis in lymphomas and other carcinomas [51]."

Minor Issues:
Figure 5c legend: Typo - "corresponding ot the structural annotation" → "corresponding to the structural annotation"

Corrected.

Extended Data Figure 2d legend: First two sentences are redundant

Corrected.

Figures 8 and 9 are not labelled

Labels are added.

Reviewer #2 (Remarks to the Author):

In this work, Li and Lehner introduce TF-MAPS (Transcription Factor-Mutational Allosteric Propagation Scan), a high-throughput method to map residues that affect

DNA binding through abundance changes, contact with DNA or allosteric effects. They implement the method to generate maps for the DNA-binding domains (DBDs) of Hepatocyte Nuclear Factor 1 homeobox Alpha (HNF1A) and two forkhead box (FOX) transcription factors (TFs), FOXG1 and FOXP1.

To evaluate the DNA binding capacity of the three DBDs, Li and Lehner employed bacterial one hybrid (B1H) using HIS3 as the reporter gene. In parallel they evaluated their cellular abundance using the spectinomycin tripartite sandwich (STS) assay. By utilizing deep mutational scanning approach, most single amino acid substitution variants and nonsense variants were screened. To disentangle changes in DNA binding due to abundance from changes due to a role in DNA binding they used LOESS (Locally Estimated Scatterplot Smoothing) regression to estimate the expected changes in binding due to abundance and then calculated the residuals between the expected and observed binding of each variant to deduce their contribution in binding independent of their abundance.

They find that the binding residuals are largest for the residues in contact with DNA and decay exponentially with increasing distance from the DNA but also vary significantly based on the kind of amino acid substitution and the exact residue in the WT sequence. Based on the residuals between the expected (based on the exponential decay model) binding residual and the observed ones, they were able to map “allosteric hotspots”. They use this knowledge along with the FPocket algorithm to identify potential targets for small molecule drugs.

The approach of combining a protein abundance screen with some sort of functional screen to draw conclusions regarding the mechanism behind the phenotype of protein variants (eg Matreyek et al., Genome Medicine, 2021 - PMID: 34649609) is not novel. Similar attempts on characterizing the effect of mutations on TFs using yeast one-hybrid have also been performed before (McDonnell et al., Molecular Systems Biology, 2024 - PMID: 38849565). However, to my knowledge the combination of an abundance screen with a DNA binding screen and the accompanying computational analysis to infer which TF residues contribute to allosteric effects is indeed novel. The method presented in this work will help with the interpretation of variant effects and with the identification of druggable pockets for the design of new small molecule drugs.

Overall, this is a rigorously executed study, and the data are robust. The results and methodology are presented clearly, and careful implementation of proper statistics contribute to a high confidence in the presented results.

We thank the reviewer for their enthusiasm and very constructive suggestions.

• It is impressive and interesting to examine the data of the abundance screen with 21 different concentrations of spectinomycin. To add to it, I believe it would

significantly increase the confidence in the abundance scores if a correlation was shown between the abundance scores and some sort of low-throughput measurement of a selected few variants' abundance covering the entire range of abundance scores (eg Western blot or tagging with fluorescence protein + flow cytometry). Given the non-linear correlation of the replicates in different conditions and the Extended Data Fig. 7k, showing a non-linear correlation between the Tm change of five variants and their abundance scores, I believe it would be useful for the interpretation of the results to show how exactly the abundance scores correlate with such low-throughput measurements.

The reviewer raises an important point. To address this we have benchmarked the spectinomycin tripartite sandwich (STS) abundance assay (Abundance) using site saturation mutagenesis of five protein domains (Title are PDB IDs) with near folding energy measurements for nearly all mutations in (Tsuboyama *et al.,* Nature 2023). This data will form part of a larger mutagenesis project still in production but we copy it below to demonstrate the good correlation between in cell abundance and in vitro quantified free energy changes. We also note that abundance measurements are not expected to be linearly related to free energy changes for two-state folding proteins, where the fraction folded is a sigmoidal function of the free energy (see e.g. Faure et al. Nature 2022).

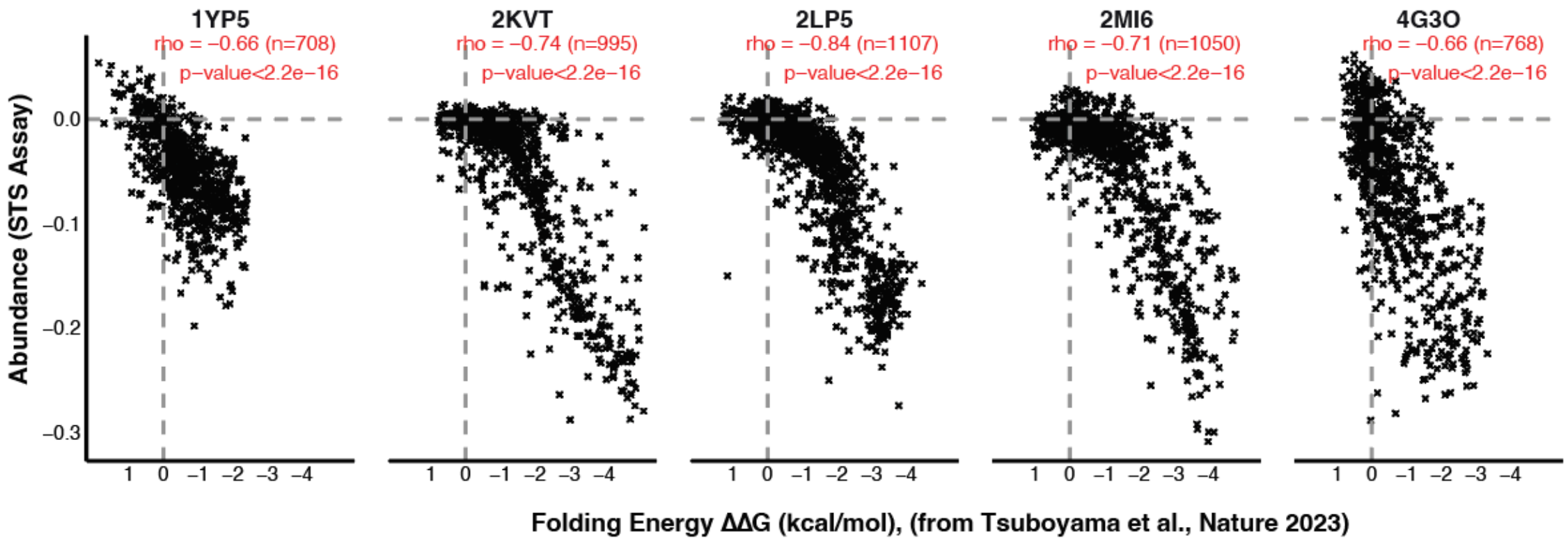

In the revised manuscript, Extended Data Fig. 1g and 6h, i, we included comparisons of STS assay results for the three TFs in our assay with ThermoMPNN (Dieckhaus et al., PNAS 2024) -predicted protein stability changes (see below):

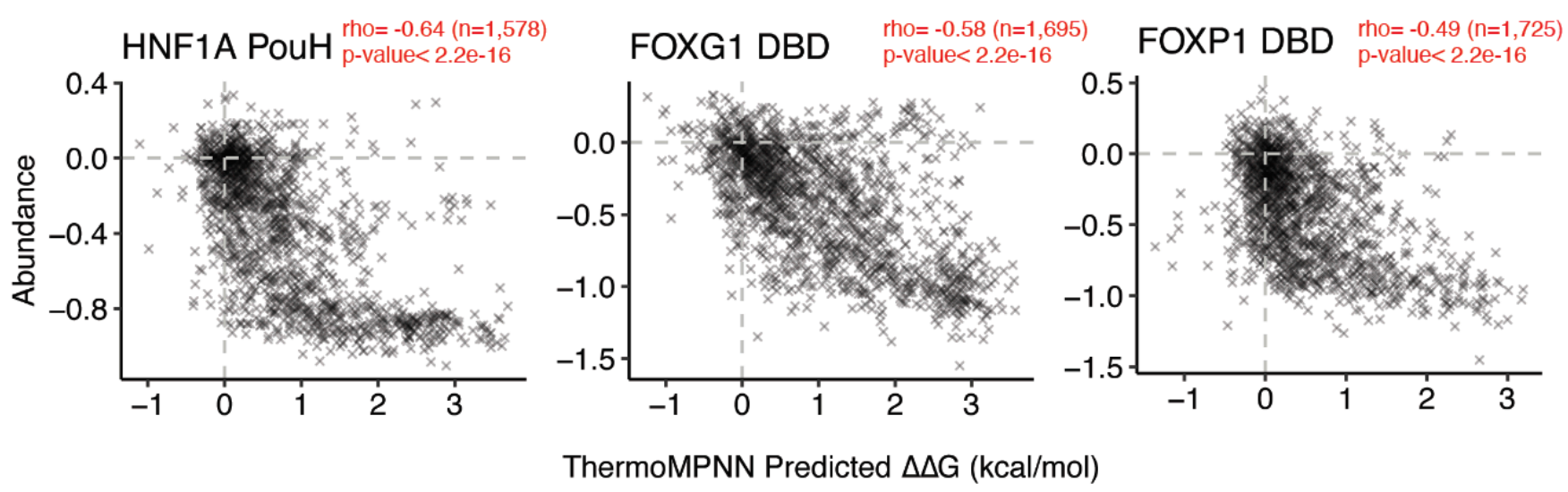

• A basic assumption used for the interpretation of the results is that mutations that change the DNA binding more than the expected (according to the change of abundance) must affect either DNA contact sites or act through some allosteric effect. How do we know that such a mutation does not increase the interaction with some endogenous protein or introduce a site of post-translational modification thus potentially inhibiting DNA binding indirectly? Perhaps this possibility should be addressed in the manuscript.

The reviewer is right about our assumption, and we cannot formally rule out the possibility that these effects on DNA binding occur indirectly, such as through interactions with endogenous proteins or post-translational modifications. Several aspects of our experimental system mitigate this likelihood, as all proteins were expressed in *E. coli,* which lacks endogenous interaction partners and most of the post-translational modification machinery present in eukaryotic systems.

We added the following information in the revised Discussion section (Line 587): "Additional mechanisms of pathogenicity exist including changes in protein localization [64,83–85], post-translational modifications [86], and alternative splicing [87] that will not be captured by our platform."

• According to the Addgene vector 18045 map, the TF DBDs appear to have been introduced at the C-terminus of the RNA polymerase subunit for the B1H screen. If that is right, could the fact that the C-terminus was exposed contribute to C-end degron effects that might not be present in the context of the STS assay, where the C-terminus is not exposed? If that is the case the interpretation of the variants near the C-terminus could drastically change when using the method in bacteria (Cragan et al., Journal of Biological Chemistry, 2025 - PMID: 40023398), yeast (Kong et al., NatureCommunications, 2023 - PMID: 38102142) or human cell lines (Koren et al., Cell, 2018 - PMID: 29779948, Voutsinos et al., biorxiv 2025 - PMID: 40463067). Could the authors discuss this possibility as a limitation of the method in this work?

We thank the reviewer for raising this important point. The reviewer is correct that in the bacterial one-hybrid (B1H) system, the transcription factor DNA-binding domain (DBD) is linked to the C-terminus of the RNA polymerase α-subunit, whereas in our spectinomycin tripartite sandwich (STS) assay, the DBD is expressed between the two fragments of the aadA gene, without an exposed C-terminal fusion partner. This architectural difference could, in principle, lead to C-terminal degron effects in the B1H context that would not be present in STS, potentially confounding the interpretation of variants near the C-terminus.

An extended unstructured region beyond the structured DBD are included in our constructs and mutations in this region do not have deleterious effects. This is shown in Fig. 2 and Fig. 7c,d. Importantly, if C-terminal exposure in B1H were introducing confounding degron effects, we would expect a subset of mutations to exhibit strong

DNA-binding defects in B1H that are not reflected in STS, resulting in systematically negative residuals (i.e., overestimation of specific DNA-binding defects). Such a pattern is not observed in our data; the residuals near the C-terminus are not systematically negative.

• Sometimes “TF” is used when referring to DBDs, especially in figures. I think for clarity it would be better if “DBD” or “TF-DBD” is used instead, given that only these domains of the TFs are screened.

Corrected throughout the manuscript, especially in the figure legend by adding DBD after TF or TF names.

• Why were the variants of very low abundance scores excluded in the calculation of the per position median absolute residuals and not simply the stop codon ones? (lines 653-655).

Thank you for this important question. We excluded variants with very low abundance scores (abundance < –0.8), rather than only stop codons, to objectively identify positions amenable to perturbation (“engineerable”) for later overlay with pockets. If all missense variants at a position fall below this threshold, the position is considered essential and unsuitable for perturbation; if at least one missense variant retains substantial abundance, we calculate a quantitative median residual from those variants that are not expected to behave like stop codons.

Importantly, these low-abundance variants were not removed from the dataset. They remain displayed in the per-pocket residual plots (Extended Data Fig. 5b and Extended Data Fig. 6c,d), where missense variants with abundance < –0.8 are clearly marked with a “/” and their individual residuals are shown.

Thus, this threshold-based filter provides a biologically meaningful and objective approach for calculating per-position medians without discarding information. We have clarified this rationale in the Methods section:

“We excluded variants with very low abundance scores (abundance < –0.8), rather than only stop codons, to objectively identify positions amenable to perturbation (“engineerable”) for later overlay with pockets. If all missense variants at a position fall below this threshold, the position is considered essential and unsuitable for perturbation; if at least one missense variant retains substantial abundance, we calculate a quantitative median residual from those variants that are not expected to behave like stop codons.”

• The bacterial species used in the study should be mentioned in the methods.

We have added the information to the methods sections ("DNA binding selections"; and "Abundance Selections"), both are *E.coli* DH5 alpha cells (Lines 718 and 731).

Minor comments:

• Line 45-46: "DNA" should go before "nuclear localisation" for the verb "binding" to refer to it.

We rephrased it to make it clearer:

"Ligands bind these TFs at a site distant to the DNA-binding interface and indirectly alter the binding of the receptors to other proteins, DNA and the nuclear localisation by causing changes in conformation and dynamics [16]."

• Line 547: Why four DBDs, aren't there only three?

This was a mistake. Corrected to three.

• Line 549: "backbone" font is different

Corrected.

• Line 681: Is "80" a typo?

Corrected. It should be -0.8

• Are references 28 and 52 the same?

Yes. Corrected.

• In Figure 1h I would change the colour of the blue rho (all) as it might be confused for the rho of the blue dots (DNA contact).

Corrected. The colour is changed to black, while the non-contact residues' rho is changed to dark grey to avoid confusion.

• In the heatmaps of Figure 2 and Figure 7 the amino acids on the vertical axes are not well aligned to the map.

Corrected.

• In Figure 6c there is a red line on the black block of the likely pathogenic and in 6d the number 43 is slightly out of the grey block.

Corrected.

• In Extended Data Figure 4a, what is the red dotted line?

A red dotted line indicating an absolute binding-residual of 0.1 was originally shown as a visual guideline. As this information is not essential, the line was removed from the revised figure to avoid confusion.

Reviewer #3 (Remarks to the Author):

The work of Li and Lehner aim to characterize the mutational landscape in transcription factors that leads to effects in binding specificity, independently of protein concentration in a high throughput manner. The methodology called Transcription Factor-Mutational Allosteric Propagation Scan (TF-MAPS), uses deep mutational scanning (DMS) and an STS assay to identify the effects of mutations on folding and not binding. After proper normalization and modeling of the expected effect of protein concentration the authors are able to compute binding-residual values that are proxy of how detrimental or enhancing is a mutation in a given residue position of a TF. The authors focus on DNA binding domains and apply their methodology in a bacterial system. Li and Lehner apply TF-MAPS to 3 different proteins and are able to verify the effect of mutations at the binding interface, estimate the decaying distant-dependent effect of mutations near the binding interface, as well as characterize those allosteric hotspots that have larger effect that expected. The authors also investigate and contrast with clinical variants and provide some mechanistic interpretation of these mutations using the binding-residual scores and its statistics as interpretative tools. Finally, the authors also identify relevant drugabble hotspots and propose some plausible sites and regions where druggability and regulation of TF binding might be possible. The authors support the idea that TF-MAPS is general and scalable as well as fast.

In my opinion, this is an interesting study that is well motivated and carefully designed to isolate the direct effects of mutation on DNA recognition by TFs. Although the use of DMS to study mutational landscapes is not new, the combination with the folded protein abundance STS assay is innovative and leads to interesting results. In particular, I appreciated the effort to validate as many mutations as possible that do have stronger residuals either via structural interpretations, literature review and modeling. This careful validation brings validity to other non-expected interpretations of the effects of mutations. I also appreciate the idea of comparing homologous TFs like FOXG1 and FOXP1, the results showcase how similar TFs that are members of the same family and conserve their interaction interfaces can have very distinct responses to mutations, showcasing the evolutionary diversity and complexity of these systems. Therefore my assessment is that both the specific results and the general methodology of TF-MAPS are valuable and has substantial contributions to the field. I see proper use of statistics and treatment of uncertainties as well as appropriate citations of prior work. My main points focus on the presentation of results and the need to do some clarifications on the limitations and

effectiveness of this technique. I will enumerate those comments first in a general sense and then give some specific points to improve the clarity of the manuscript.

We thank the reviewer for their enthusiasm and very constructive suggestions.

General Comments

1. One important aspect that needs to be clearly stated is that this methodology only considers single site mutations. Even though DMS leads to a large amount of variants, there is still a much larger combinatorial space where epistatic interactions might dominate and alter both direct binding and allosteric effects. Authors should comment on this and clarify this limitation, as the current manuscript leads to the idea that the landscapes obtained are global and “comprehensive”.

Thank you for raising this important point. We have stated in the manuscript that it is a comprehensive map of single amino acid substitutions.

Line 96:
“We first use TF-MAPS to chart a comprehensive map of single amino acid substitutions in the POU-Homeodomain (POU_H) DNA-binding domain (DBD) of Hepatocyte Nuclear Factor 1 homeobox Alpha (HNF1A).”

2. The title of the manuscript as well as the name of the methodology highlights the concept of allostery, yet a big part of the results focus on direct effect on the TF-DNA interface. Although important for validation, I left thinking that more analysis or focus should be done to the allosteric hotspots. For example, I am curious if there are any allosteric hotspots that have a larger residual effect than interfacial mutations and if so, a potential interoperation might be valuable.

We added more information regarding allosteric hotspots in response to the reviewer’s comment. To answer the reviewer’s question directly about whether there are any allosteric hotspots larger than interfaces, we ordered the residues by their median absolute binding residuals (as shown below & revised Extended Data Fig. 4f; Extended Data Fig. 7g).

For HNF1A, Line 310:

“None of these allosteric hotspot residues have larger effects than the median of residues in the DNA-binding interface (Fig. 4b; Extended Data Fig. 4f)”

For FOXG1 and FOXP1, Line 424:

“Unlike the HNF1A POU_H domain, six surface and core residues in the DBDs of FOXG1 (R244, P182, R201, E209, G250, and Q226, listed from largest to smallest

residual) and two core residues in FOXP1 (L473 and F541) show median absolute binding residuals larger than half of those observed at DNA-contacting interfaces (Extended Data Fig. 7g). Two FOXG1 residues (R244 and G250) exhibit negative median binding residuals, whereas the remaining four (P182, R201, E209, and Q226, listed from largest to smallest residual) show positive median binding residuals. In FOXP1, L473 shows negative binding residuals, while mutations at F541 lead to positive residuals.

None of these residues are aligned positions between the two DBDs. These six residues in FOXG1 are spatially dispersed (Fig. 7i) and the two FOXP1 core residues (L473 and F541) reside in the two helices, which are folded closely together (Fig. 7j). ”

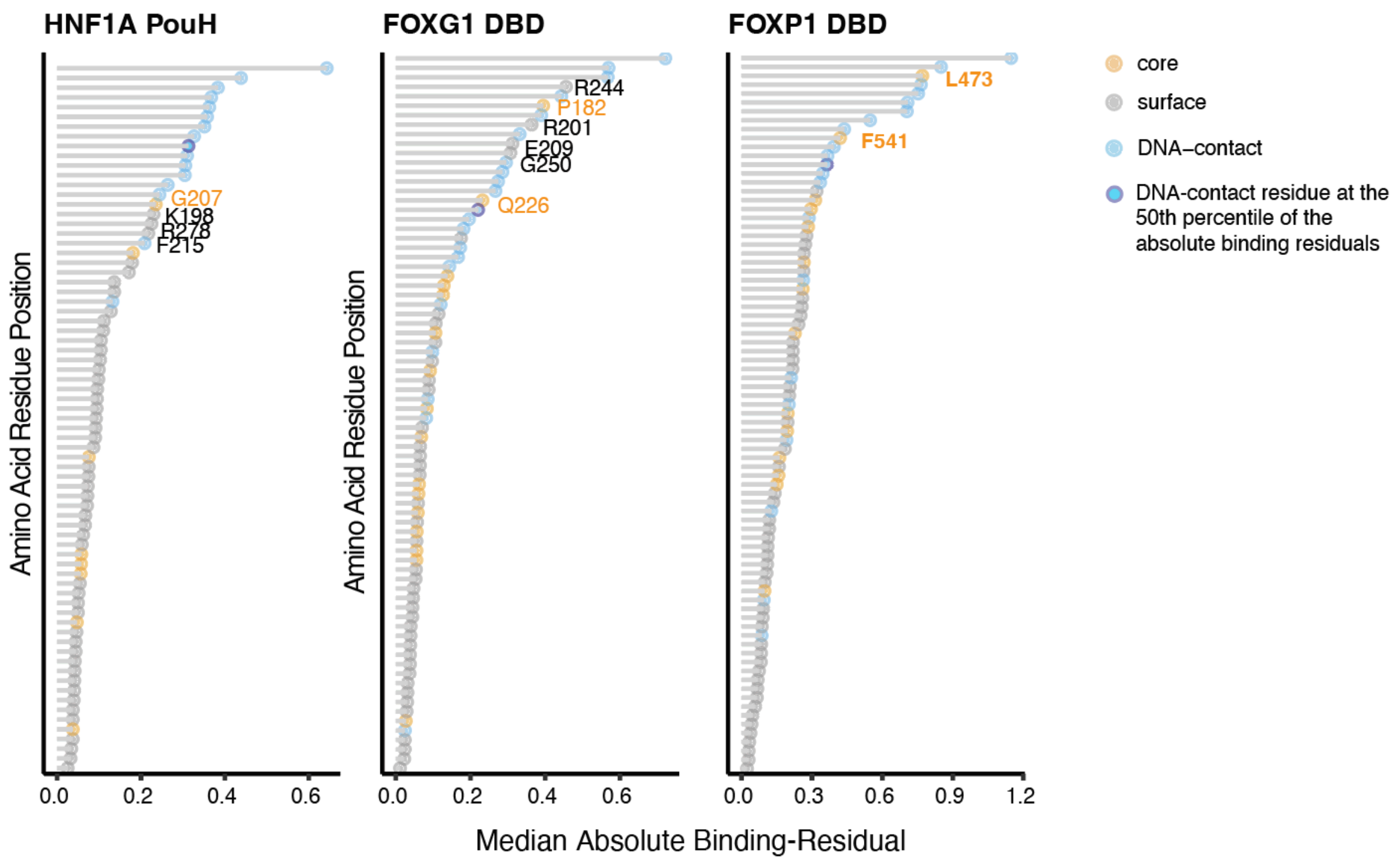

3. Many transcription factors have allosteric communication between a sensing domain and the DBD that activates/deactivates them. Why did the authors not include this in their work? The authors do mention that in the future it would be valuable to include more TF domains, however, I feel that the more interesting cases of allostery are found when a sensing domain binds to a ligand that later induce DNA recognition. The authors should justify why this was not part of their investigation and provide maybe a putative analysis of its importance.

Our primary motivation, as stated in the introduction (Lines 39-67), was to evaluate allostery in TFs that do *not* have known regulation by allosteric ligands. This is the vast majority of human TFs.

4. Although it is evident that the authors did a comprehensive study of mutations, I feel their figures are too crowded. Many times the information needed to convey their message as explained in the text requires only a fraction of the data shown in figures. I suggest the authors to move redundant or non-discussed data on figures to the SI.

In response to the reviewer's comment, we reduced the figure panels in this revised manuscript. The original Fig. 3 panels b, e; Fig. 4 panels f, g; Fig.7 panels a and b were moved to Extended Data Figures.

5. The authors highlight the "fast" nature of TF-MAPS. One argument provided is the fact that this is done in a bacterial system. However, it is not clear to me why this high throughput methodology is faster than other types of DMS based systems. Given that this is important for adoption, I suggest that the authors spend more time explaining or comparing why TF-MAPS is faster or more efficient than other methodologies.

We have added the following text to the Introduction (Line 179):

"However, because TF-MAPS is conducted entirely in bacteria, it offers the advantages of speed, simplicity, and smaller volumes, making it more scalable. From transformation to completion, our bacteria-based assay takes less than 2 working days (e.g., starting at 3 pm and finishing before noon the following day). This is substantially faster than yeast and mammalian-based selections. The selection volumes are also much smaller."

Specific Comments

1. Page 6, line 191. The authors mention that the HNF1A binding of each variant against its abundance shows a strong non-linear correlation. I believe the wording is a bit confusing as it makes it sound like the correlation is strongly non-linear. I believe the authors refer to a strong correlation. Looking at the data, I don't see a clear non-linearity and I don't think it affects conclusions so I would probably only refer to it as a correlation or dependence. If the authors think the non-linearity is important then there are ways to quantify the non-linearity, one way is contrasting Spearman with Pearson correlations and show a big deviation.

We agree and have re-worded this to "strong correlation".

2. Fig.1a is clean and simple but a bit hard to follow, I suggest to improve it for clarity.

We have modified Fig. 1a to include additional information on what to expect from mutations affecting folding and binding, respectively, in each assay.

3. I am not sure if the term “strict contacts” is the most appropriate for contacts based on Alphafold3, since those complexes are coming from a predictive algorithm. I would think that those coming from experimental structures should be called “strict” and those from AF3 perhaps “estimated”? Also in page 10, there is a reference to “direct contacts”, which makes this a bit more confusing. Perhaps I am missing something here, in any case it would be good to clarify.

We agree and have revised the text throughout the manuscript to clarify this distinction, referring to "Curated Contacts" (for DNAproDB criteria) and the remaining “∆rSASA-based” contacts. We apply this identically to experimental and AlphaFold3 models because they exhibit high structural concordance (Extended Data Fig. 3) and thus yield the same curated contact set regardless of source. DNAproDB is intentionally conservative: it requires hydrogen bonding, van der Waals overlap, or burial of surface area, yielding a higher-confidence contact set but being stricter than a simple ∆rSASA-based or distance-based cutoff.

4. For the predictive druggable pocket section, and more importantly for the ones with higher druggability scores, please provide a table or data file with the residues involving the pocket.

We have added the residue-level composition for all predicted pockets to the revised Supplementary Table. The complete dataset, including comprehensive per-residue information, has been deposited in Zenodo (https://zenodo.org/records/17457956) and is now cited in the Data Availability statement.

5. Page 13, line 480. Change “surface allosteric surfaces” with “allosteric surface”.

Done

6. Page 16, Line 593. A single sentence paragraph is non-standard, I suggest merging with the previous paragraph.

Done
